# Transformation of microbiology data into a standardised data representation using OpenEHR

**DOI:** 10.1038/s41598-021-89796-y

**Published:** 2021-05-18

**Authors:** Antje Wulff, Claas Baier, Sarah Ballout, Erik Tute, Kim Katrin Sommer, Martin Kaase, Anneka Sargeant, Cora Drenkhahn, Infection Control Study Group, Patrick Fehling, Sabine Rey, Markus Suhr, Vanessa M. Eichel, Nico T. Mutters, Klaus Heeg, Petra Gastmeier, Michael Behnke, Luis Alberto Peña Diaz, Sylvia Thun, Roland Eils, Alexander Mellmann, Hauke Tönnies, Benedikt Zacher, Tim Eckmanns, Timo Sztyler, Brandon Malone, Angela Merzweiler, Martin Dugas, Michael Storck, Marcel Wunderlich, Tatiana von Landesberger, Benjamin Gebel, Thorsten Klingen, Stephan Glöckner, Dirk Schlüter, Michael Marschollek, Simone Scheithauer

**Affiliations:** 1 Peter L. Reichertz Institute for Medical Informatics of TU Braunschweig and Hannover Medical School, Carl-Neuberg-Str. 1, 30625 Hannover, Germany; 2 Institute for Medical Microbiology and Hospital Epidemiology, Hannover Medical School, Carl-Neuberg-Str. 1, 30625 Hannover, Germany; 3 Institute of Infection Control and Infectious Diseases, University Medical Center Göttingen (UMG), Georg-August University Göttingen, Robert-Koch-Str. 40, 37075 Göttingen, Germany; 4 Institute of Medical Informatics, University Medical Center Göttingen (UMG), Georg-August University Göttingen, Robert-Koch-Str. 40, 37075 Göttingen, Germany; 5 IT Center for Clinical Research (ITCR-L) and Institute of Medical Informatics (IMI), University of Lübeck, Ratzeburger Allee 160, 23538 Lübeck, Germany; 6 Section for Hospital Hygiene and Environmental Health, Centre of Infectious Diseases, Heidelberg University Hospital, Heidelberg, Germany; 7 Institute for Hygiene and Public Health, University Hospital Bonn, Bonn, Germany; 8 Medical Microbiology and Hygiene, Department of Infectious Diseases, Heidelberg University Hospital, Heidelberg, Germany; 9 Institute of Hygiene and Environmental Medicine, Charité - Universitätsmedizin Berlin, Berlin, Germany; 10 Berlin Institute of Health (BIH), Berlin, Germany; 11 Charité - Universitätsmedizin Berlin, Berlin, Germany; 12 Hochschule Niederrhein - University of Applied Sciences, Krefeld, Germany; 13 Institute of Hygiene, University Hospital Muenster, Muenster, Germany; 14 Robert Koch Institute, Berlin, Germany; 15 NEC Laboratories Europe GmbH, Heidelberg, Germany; 16 Department MIS, Heidelberg University Hospital, Heidelberg, Germany; 17 Institute of Medical Informatics, University of Muenster, Muenster, Germany; 18 Technical University of Darmstadt , Darmstadt, Germany; 19 Department of Infectious Diseases and Microbiology, University Medical Center Schleswig-Holstein, Luebeck, Germany; 20 Helmholtz Centre for Infection Research (HZI), Braunschweig, Germany

**Keywords:** Microbiology, Computational biology and bioinformatics

## Abstract

The spread of multidrug resistant organisms (MDRO) is a global healthcare challenge. Nosocomial outbreaks caused by MDRO are an important contributor to this threat. Computer-based applications facilitating outbreak detection can be essential to address this issue. To allow application reusability across institutions, the various heterogeneous microbiology data representations needs to be transformed into standardised, unambiguous data models. In this work, we present a multi-centric standardisation approach by using openEHR as modelling standard. Data models have been consented in a multicentre and international approach. Participating sites integrated microbiology reports from primary source systems into an openEHR-based data platform. For evaluation, we implemented a prototypical application, compared the transformed data with original reports and conducted automated data quality checks. We were able to develop standardised and interoperable microbiology data models. The publicly available data models can be used across institutions to transform real-life microbiology reports into standardised representations. The implementation of a proof-of-principle and quality control application demonstrated that the new formats as well as the integration processes are feasible. Holistic transformation of microbiological data into standardised openEHR based formats is feasible in a real-life multicentre setting and lays the foundation for developing cross-institutional, automated outbreak detection systems.

## Introduction

Spread of multidrug resistant organisms (MDRO), for instance Carbapenem-resistant *Klebsiella pneumoniae*, can be driven by nosocomial transmission within hospitals and even between different hospitals^1^. The emergence of MDRO may be caused by sporadic transmission events or in the context of a nosocomial outbreak involving several patients^2–4^. When (nosocomial) infections are caused by MDRO, timely and appropriate anti-infective therapy is a challenge and patient mortality can be increased^5^. Thus, it is crucial to prevent spread (transmission) of MDRO in hospitals. Looking at surveillance systems one pivotal and well accepted criterion is the timely detection of relevant events such as a single highly relevant transmission event or a beginning nosocomial cluster to timely implement control measures^6^. The tracing of patients with MDRO and susceptible bacteria in hospitals for infection control reasons is time-consuming and complex since numerous microbiology data sets have to be monitored often manually for a certain period of time.

In times of emerging digitalization in healthcare, it seems obvious to develop data-driven algorithms that are able to automatically detect nosocomial clusters^7,8^. Thus, relevant information can be provided for the clinical staff at the right time and clusters can be stopped before developing outbreaks. Such approaches require availability and accessibility of data in a high quality manner. In particular, for outbreak control, the synthesis of both clinical microbiologic laboratory data and fine granular patient movement data is crucial to detect, analyse and finally interrupt transmission pathways. However, although the overall amount of data is increasing, harmonizing these data sets (which are often generated in various hospital information systems and applications without any interconnections, standardized definitions and open interfaces) remains challenging^9^.

In the *HiGHmed Infection Control* project, which is funded by the German Federal Ministry of Education and Research within the German Medical Informatics Initiative^10^, we aim at developing an automated, algorithm-based and open source application called Smart Infection Control System (SmICS) for prospective outbreak detection in hospitals^11^. This tool shall be used for supporting infection control specialists in daily decision-making regarding infection control. In principle, such system can then be used for monitoring MDRO, susceptible bacteria as well as viruses (such as influenza or SARS-CoV-2). We strive for designing such application to operate cross-institutional on a national and international level. Therefore, we first need to standardize and harmonize the required data, such as the microbiology findings, to reach unambiguous and consented data models across local contexts.

As this is a matter of particular interest beyond the described *Infection Control* use case, the overall goal of HiGHmed as a nationwide data infrastructure project is the establishment of an interoperable and open health data platform that will enable the efficient reuse of routine data from research and clinical care to foster cross-institutional data access, analytics and sharing^9^. To make data interoperable, traceable, accessible and reusable in such a platform, data need to be clearly defined, modelled and integrated in close cooperation between computer scientists and clinical experts using a standardized, internationally accepted modelling approach. In HiGHmed, openEHR^12^ is used as standard for semantic data modelling, enriched by the use of international terminologies such as LOINC^13^ and SNOMED CT^14^.

In this work, we present the results on transforming microbiology data from commercial primary source systems into standardized, consented data models by using openEHR. Furthermore, to demonstrate the reusability potentials of these newly represented and standardized microbiology data sets, we present an openEHR based application as proof-of-principle. This work will lay the foundations for developing further standardized and interoperable applications such as the above mentioned SmICS.

## Methods

### Clinical information modelling using OpenEHR

We adopt the openEHR approach for semantic modelling as openEHR relies on a reference model clearly separating technical and domain content^12^. This multi-level modelling is realized by providing a reference model that defines the technical-driven aspects of developing databases, application systems or electronic health records, and a formal but domain-driven definition of concepts in the form of so-called *archetypes*. Archetypes allow the strict and unambiguous description of clinical concepts such as a laboratory test or a blood pressure measurement. Each archetype is an extensive collection of attributes that in the end form a maximum set of relevant data in any conceivable use case in need of this concept. Although archetypes are designed in close cooperation with clinical experts and without consideration of the technical implementation of the underlying persistence layer, they are subject to a formal description language called Archetype Definition Language (ADL)^15^. First, the general purpose of an archetype need to be specified by setting the archetype class to COMPOSITION, OBSERVATION, INSTRUCTION, ACTION, or CLUSTER. Each class comes with different features and delivers a so-called ‘data’ feature allowing the representation of the key information on the clinical model, e.g. the systolic and diastolic values of a blood pressure measurement. However, for some classes there are specific features available, e.g. the OBSERVATION class contains a ‘state’ feature able to capture any further data that is needed for the correct interpretation of the content. An example is the position of the patient (e.g. lying, sitting) during a blood pressure measurement. Furthermore, e. g. the ACTION class contains a ‘pathway’ feature to identify the state of the action as a part of a clinical process (e.g. therapy started, therapy ended).

Each archetype item is identified by a unique path that can be accessed to retrieve data from this archetype. For querying data, these paths are used within the dedicated query language called Archetype Query Language (AQL)^16^. AQL is a query language that acts upon the archetype and not the database level meaning that each AQL query is executable in any openEHR based data repository as long as the same archetypes are used. To ensure semantic enrichment of the models, archetypes can be bound to external terminologies^17^. It is possible to bind terminologies such as LOINC or SNOMED-CT at the level of the concept (e.g. blood pressure bound to SNOMED CT: 75367002 | Blood pressure (observable entity)), at the level of data elements (e.g. systolic value bound to SNOMED-CT: 271649006 | Systolic blood pressure (observable entity)), or at the level of values (e.g. raised blood pressure bound to SNOMED CT: 260399008 | Raised (qualifier value)). Furthermore, it is possible to define coded value lists for an archetype item by using local terms. For designing archetypes, various modelling tools are available^18^. In our project, we used the Archetype Editor and Template Designer (by Ocean Informatics in London, UK and Chatswood, AU) as well as the ADL Designer (by Better d.o.o. in Ljubljana, SL).

Archetypes are modelled in various projects all over the world which is why an international archetype repository was set up to maintain the international archetype creation. The international Clinical Knowledge Manager (CKM)^19^ as a web-based tool that is freely accessible and openly available is used to upload, review, govern, maintain and publish archetypes internationally to enable reuse of existing archetypes. However, some local instances of the CKM are also available such as our HiGHmed CKM^20^. In our project, we use the HiGHmed CKM because although the archetypes are managed in the international CKM, they can be referenced through a direct link. Thus, we can work with our local archetypes and the international models in the same tool. Each archetype carries a publication status according to its version such as *draft*, *under review* or *published*. A published archetype is at least in version 1.0; however, it only reaches this status after going through several so-called *review rounds*. All publication states are visible in the CKM.

To represent specific use cases, for example discharge letters or laboratory reports, archetypes can be composed to define so called *templates*. Hereby, archetypes can be nested through the use of so-called ‘slots’ and can also be constrained: items that are not relevant in the specific context can be hidden, cardinalities can be restricted, and allowed values can be bound to a coded value list or a restricted terminology subset.

The use of standardized models that are enriched with terminologies is considered as important prerequisite for successful integration of data into an open health data platform. Various implementations of the openEHR reference model are available that can be used as openEHR based data repository to store the harmonized, standardized and integrated data^21–24^. For our project, we use the *better platform* by Better^25^.

### Clinical knowledge governance

**Figure 1:**
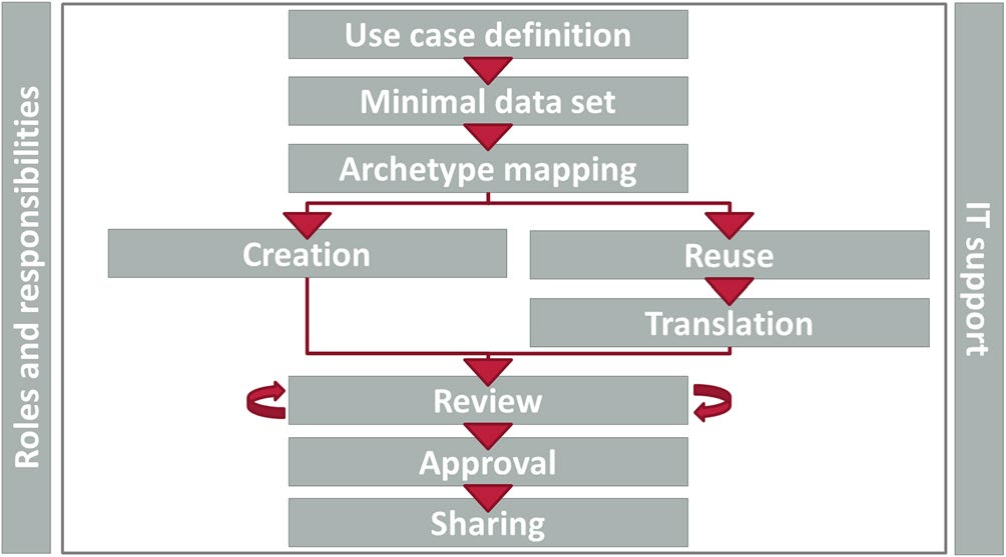
Clinical Knowledge Governance in HiGHmed (core modelling workflow).

All models that need to be designed must be jointly developed and agreed between computer scientists and clinical (domain) experts in predefined modelling and review processes. For HiGHmed, we developed a *Clinical Knowledge Governance Framework* defining modelling workflows, roles and responsibilities, and IT tools. The core modelling process (see Fig. 1) consists of the following steps.

In cooperation with clinical experts, a minimal data set was defined. Data were organized into categories and mapped onto archetype classes. To promote archetype sharing beyond HiGHmed, existing archetypes from international archetype repositories such as the international CKM were reused. For any non-existing concept, archetypes were designed from scratch. To be able to design new archetypes, research in the context of MDRO, interviews with medical experts and observations of the routine processes in infection control and MDRO monitoring in different institutions have been carried out. Review rounds with domain experts have been conducted by dedicated persons called *data stewards* to achieve consensus on the content and translation of archetypes. Accepted archetypes can be used for data integration, and will be returned to the global community. Our modelling group for the Infection Control project consists of five data stewards and eight domain experts from six university hospitals in Germany. For further details on the Clinical Knowledge Governance Framework, we refer to Wulff et al.^26,27^.

### Medical Data Integration Centres and HiGHmed platform

The interoperable health data platform evolving within the HiGHmed project is realized by establishing so-called medical data integration centres (MeDIC) at each participating university medical centre. In HiGHmed, data from clinical routine, clinical research and external data sources are harmonized and integrated into data repositories provided by these local MeDICs^9^. Patient-related data such as movement data, laboratory data or other parameters (e.g. outcome parameters such as death) which are electronically accessible can be integrated as soon as consented, standardized data models are available.

Currently, eight sites from Germany participate in the HiGHmed project and set up such a MeDIC. Six of these sites contribute to the Infection Control use case of this project (there are also other use cases, e.g. from Cardiology and Oncology) and transform German microbiology data from their primary source systems into their local data repository (using the same consented, in this paper presented, data model for representation of microbiology reports, amongst others). The MeDICs are based on a generic architecture that follows the basic principles of open service interfaces, information models and system specifications. The scalable framework architecture of the MeDICs is based on open and well-defined standards to enable reliable processing of data in local and distributed contexts. This ensures the integration of new data sets as well as new institutions in the long term. Extracted data from care and research remain in the respective institution or MeDIC, but are made accessible cross-institutionally with respects to data protection and security regulations^9^. This creates a consistent platform for the development of clinical application and research systems based on harmonized data sets from health care and research, which can be used across institutions and can retrieve data from other sites in accordance with data protection regulations. Consequently, if participating institutions decide to do so, it will be possible to exchange data between different institutions/clinics (national and international) in a standardised way.

The primary source systems relevant for the Infection Control project vary between the participating institutions; e.g. for the Hannover Medical School, M/Lab (Dorner, Müllheim, Germany) is used as documentation system for microbiology results (laboratory information system). In Hannover, we used extraction, transformation and load (ETL) tools and approaches that were used for similar research projects before^28,29^. Although the integration process will differ between the institutions, in the end, all data sets will be available at every local site in an openEHR based data repository in the same format preserving the semantic meaning of the data.

### Evaluation

We evaluate the correctness and quality of the transformed microbiology data sets by (1) comparing ten microbiology reports, transformed into the new standardized format and visualized in the openEHR based proof-of-principle application, with the original reports from the primary source system, and by (2) automatically calculating simple statistics on two years data sets by using an openEHR based data quality tool.

1. Comparison
  Ten reports with different complexity were selected independently by two infection control experts. For each report, pre-defined items, e.g. the total number of pathogens found or the detailed antibiogram of the bacteria (see Supplementary Information, Supplementary Data 1) were defined by the infection control experts as ground truth by using the non-transformed, original microbiology reports. In parallel, and without knowing the experts’ results, the selected reports were queried and visualised in the transformed and standardized format from the new data repository by using the proof-of-principle application. The results were compared per report. Discrepancies were discussed and documented.
2. Data quality check
  To evaluate whether the data integration process is successful even for larger data sets, we integrated the microbiology reports (bacteriology) from two years at one participating centre. By using an openEHR based data quality tool, openCQA^30^, we were able to gain an overview of the amount and the validity even of a large set of integrated data. We specified the data of interest as AQL queries in openCQA, which retrieved the data from standardized interfaces of the HiGHmed platform and automatically generated so-called *measurement methods*. These measurement methods are adaptable R-scripts^31^ each generating certain information about the quality and validity of the data set, e.g. statistical measures or plots describing the data. Using openCQA, we applied these R-scripts on the transformed microbiology data set. We checked whether the results matched the expectations for valid data (so called ground truth). The ground truth results for the statistical calculations were available from regular elaborate manual data analysis carried out by other domain experts responsible for hygiene and microbiology controlling. In particular, we defined three tasks that should be solved with the tool including:
  Proportion of *Methicillin-resistant Staphylococcus aureus* (MRSA): Percentage of MRSA among all *Staphylococcus aureus* isolates.
  Proportion of Meropenem-resistant *Klebsiella pneumoniae*: Percentage of Meropenem-resistant strains among all *Klebsiella pneumoniae* isolates.
  Quantitative analysis: Distribution of bacterial counts in positive urine samples with *Escherichia coli* as the sole pathogen.

### Ethics approval

The project was approved by the local ethics committees of the participating sites [Ethics Committee of the Hannover Medical School, no. 9245_BO_K_2020]. All research presented in this manuscript was performed in accordance with applicable relevant guidelines and/or regulation.

### Consent to participate

Informed consent was waived by the ethics committee and the data commissioner [Ethics Committee of the Hannover Medical School, no. 9245_BO_K_2020]. Informed consent is not needed because the study is based on the German Infection Protection Act (‘*Infektionsschutzgesetz’*, IfSG; German) and the national hygiene regulations (*‘Landeshygieneverordnungen’;* German)*.*

## Results

### Minimal data set

As a first step, a set of data items required to represent a microbiology report was defined collaboratively between all participating institutions (*minimal data set*). In this paper, we focus on the modelling and integration of the microbiology data. However, also a minimal data set for general patient data and the representation of movement data (e. g. patient admission, discharge and all locations the patient visited during a hospital stay) were defined for the future work towards the SmICS application.

In our minimal data set for microbiology data, the microbiology report contains different sections on

1. the report metadata,
2. the specimen,
3. the bacteria found or not found, and
4. the antibiogram of the bacteria identified.

### Data models: archetypes

In total, nine archetypes were used to represent all data items from the minimal data set for microbiology. We decided to use archetypes that are available in the international CKM. However, we needed to model three archetypes from scratch in collaboration with medical experts from the participating institutions because no international concept was available. There were no archetypes available to represent *pathogen details*, *(laboratory) locations* and *case identification of the patient’s hospital stay (episode of care)*. Consequently, our reusability rate can be reported as 67%. All archetypes used are listed in Table 1 and can be found in the CKM.

**Table 1:** Overview of archetypes used for representing microbiology data.

Concept	Name	Internationally published?
Result report	openEHR-EHR-COMPOSITION.report-result.v1 https://openehr.org/ckm/archetypes/1013.1.1324	Yes
Case identification	openEHR-EHR-CLUSTER.case_identification.v0 https://openehr.org/ckm/archetypes/1013.1.567	No, local CKM draft
Laboratory test	openEHR-EHR-OBSERVATION.laboratory_test_result.v1 https://openehr.org/ckm/archetypes/1013.1.2191	Yes
Specimen	openEHR-EHR-CLUSTER.specimen.v0 https://openehr.org/ckm/archetypes/1013.1.331	CKM review phase
Anatomical location	openEHR-EHR-CLUSTER.anatomical_location.v1 https://openehr.org/ckm/archetypes/1013.1.587	Yes
Laboratory test analyte	openEHR-EHR-CLUSTER.laboratory_test_analyte.v1 https://openehr.org/ckm/archetypes/1013.1.2881	Yes
Laboratory test panel	openEHR-EHR-CLUSTER.laboratory_test_panel.v0 https://openehr.org/ckm/archetypes/1013.1.2192	Yes
Pathogen details	openEHR-EHR-CLUSTER.erregerdetails.v1 https://ckm.highmed.org/ckm/archetypes/1246.145.322	No, local CKM published
Location	openEHR-EHR-CLUSTER.location.v1 https://ckm.highmed.org/ckm/archetypes/1246.145.801	No, local CKM published

**Figure 2:**
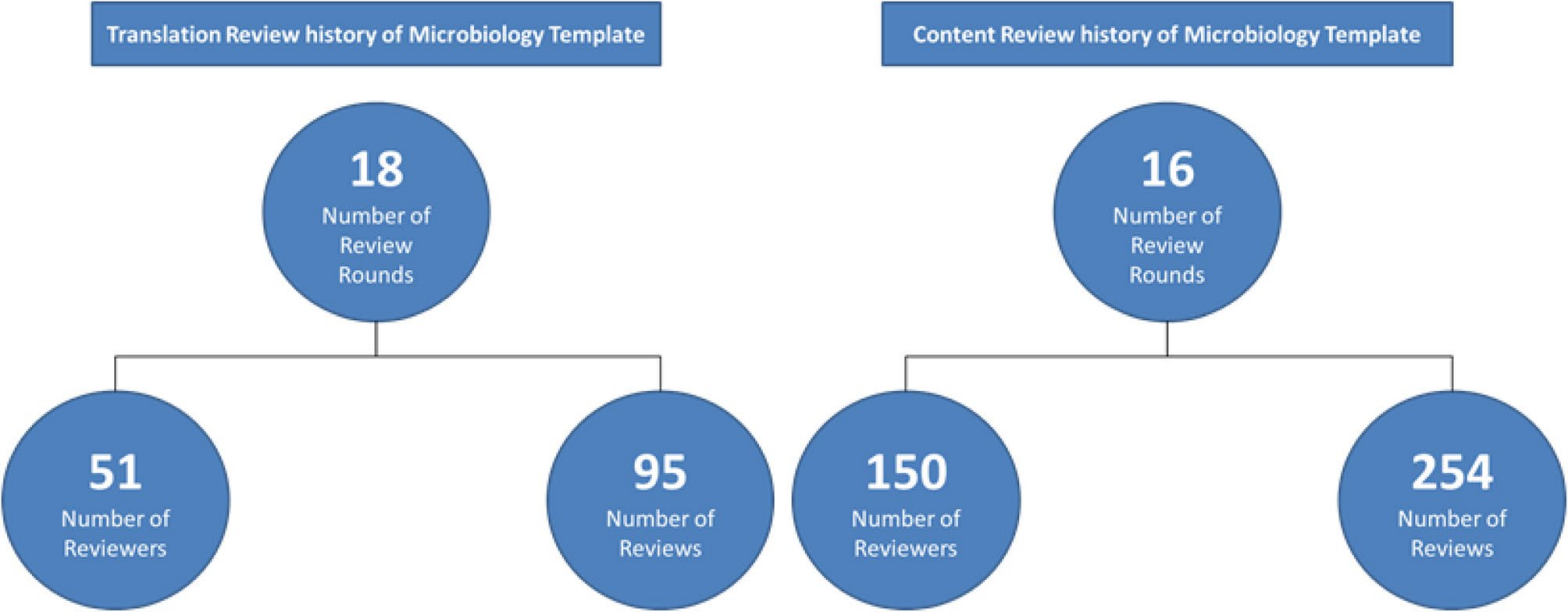
Review history of microbiology template.

Each archetype carries a publication status. From the reused archetypes, five of our selected archetypes are internationally published and one is still under review. We managed to get two of our three self-modelled archetypes published locally. In total, we conducted 16 content review rounds with 150 reviewers from the participating university medical centres and 254 reviews (see Fig. 2). For the translation of reused archetypes, 95 translation reviews by 51 reviewers were conducted in 18 review rounds.

### Data models: template

The above-mentioned archetypes were combined and nested to represent a microbiology report. The structure of the template can be described as follows. The *COMPOSITION.report-result* acts as the container of all relevant data of the microbiology examination. Furthermore, some metadata such as the report identification, the state of the report (final or preliminary) and the associated case number of the patient’s hospital stay are described within the *result report*. Each report carries one laboratory finding represented as the *OBSERVATION.laboratory_test_result* archetype. The archetype slot *specimen detail* is filled with a specimen description (*CLUSTER.specimen*), including the specimen type, laboratory specimen identifiers, timestamps of when the specimen was collected and received, comments of the collector and the anatomical location from where the specimen was taken. For the latter, the *CLUSTER.anatomical_location* is nested within the corresponding slot. The overall laboratory test result archetype also carries the detailed test result by nesting the archetype *CLUSTER.laboratory_test_panel* within the test result slot. The laboratory test panel records all microorganism findings together in a panel structure. For representing each microorganism finding individually, the archetype *CLUSTER.laboratory_test_analyte* is used. For each microorganism, the complete subtree will be filled with data.. In our data model, the term ‘analyte’ in a microbiology report is interpreted as ‘growth of any bacteria’ or ‘no growth of no bacteria’. Only in some cases (e.g. screening for MDRO), a specific microorganism is searched for. In any case, the overall test request name is recorded within the protocol section of the *OBSERVATION.laboratory_test_result* archetype (e.g. test requested: MRSA). Furthermore, here, the details of the requesting and the receiving laboratory are captured.

**Figure 3:**
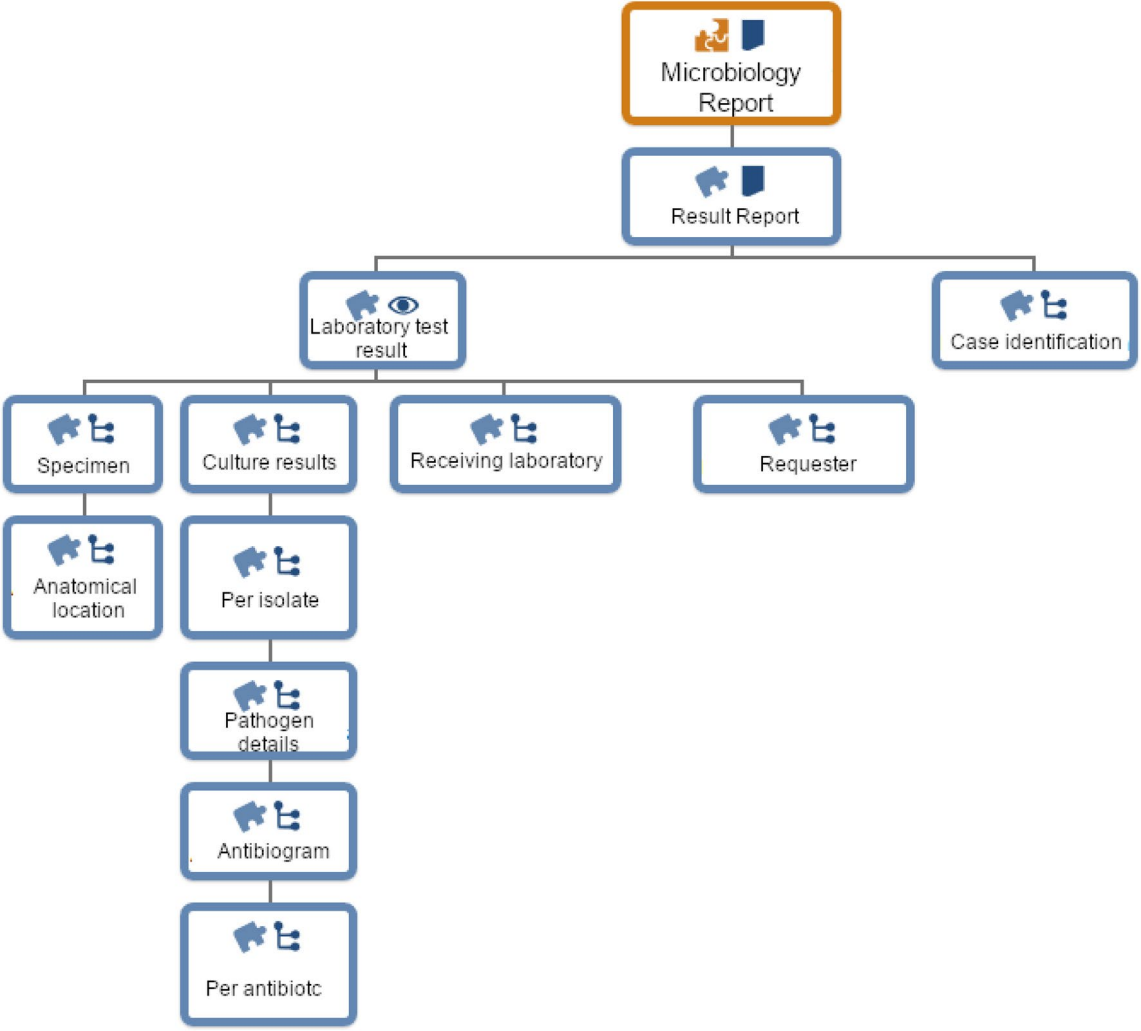
Overview of the hierarchy structure of the openEHR template for microbiology results.

For each bacterium identified, the presence or absence attribute is filled together with the strain’s concrete name, the isolate number and further comments. By using our self-modelled archetype *CLUSTER.pathogen_details*, we are also able to represent the sub type of the bacterium together with potential virulence factors, resistance characteristics and its MDRO class if applicable (e.g. MRSA). Furthermore, if available, the bacterial count (quantitative and semi-quantitative) can be stored. For capturing the details of an available antibiogram, we again reused the *CLUSTER.laboratory_test_panel* archetype together with the CLUSTER.laboratory_test_analyte archetypes to represent the collection of all antibiotics evaluated against the bacteria identified. For each antibiotic substance, the name, the minimal inhibitory concentration, the susceptibility defined e.g. by EUCAST (European Committee on Antimicrobial Susceptibility Testing) and some additional comments can be filled. The representation of the susceptibility is not restricted to a specific definition such as EUCAST but can include different definitions. The definition used will be stored together with the concrete values of the resistance pattern to assure interpretability. The structure of the template is presented in Fig. 3.

### Data models: terminologies and value lists

To assure a broad reuse of archetypes, it is recommended to set terminology bindings at template level. Usage of terminologies enables future advanced, machine-based evaluations. SNOMED CT in particular is built upon a foundation of description logic resulting in rich computer-processable definitions and hierarchies. Once information is represented via SNOMED CT, these structures facilitate the aggregation of similar entries and the discovery of related concepts. In terms of infection control, these features can improve outbreak detection by recognizing pathogens as part of the same cluster even though they are represented with slight discrepancies, e.g. in different granularities.

In total, we defined 12 value sets from which seven were mapped to international terminologies such as SNOMED CT. Some of the defined value lists are not final and not standardized instances are still allowed. This is due to the fact that, at first, only instances defined as *mandatory* in the minimal data set discussions, needed to be standardized across the participating institutions. However, a further enhancement is aspired. The Supplementary Information, Supplementary Data 2 shows the consented value lists and selected terminologies.

To outsource the complexity of terminology interaction from within applications, the HiGHmed consortium decided to license a terminology server: *Ontoserver* by CSIRO^32^ which is based on the emerging FHIR standard^33^ and enables various valuable functions. Coding systems and value sets needed are agreed upon in the use case infection control and uploaded to the Ontoserver as FHIR resources. Afterwards, these can be accessed (amongst others) to validate data used in applications, to automatically map local, proprietary codes to standardized terminologies and—as described above—to query advanced content and relations.

### Data integration

All participating sites transformed original data available in different relational representations into the consented openEHR template based representation. For example, in Hannover, we decided to evaluate our local data integration approach by integrating all bacteriologic reports from two years that are available in a commercial laboratory information system for microbiology results (here M/LAB). 260.084 reports from 51.947 patients were successfully transferred to the openEHR based representation. As another example of data integration, Göttingen successfully integrated 3.5 million retrospective microbiological data sets (from three years) and 382 real time microbiological data sets (from one month) into an openEHR repository using the same consented data models.

### Applications and data queries

Based on the standardized data repository many different data queries can be tailored and interoperable applications^29^ can be developed demonstrating the manifold possibilities of reusing the transformed data.

**Figure 4:**
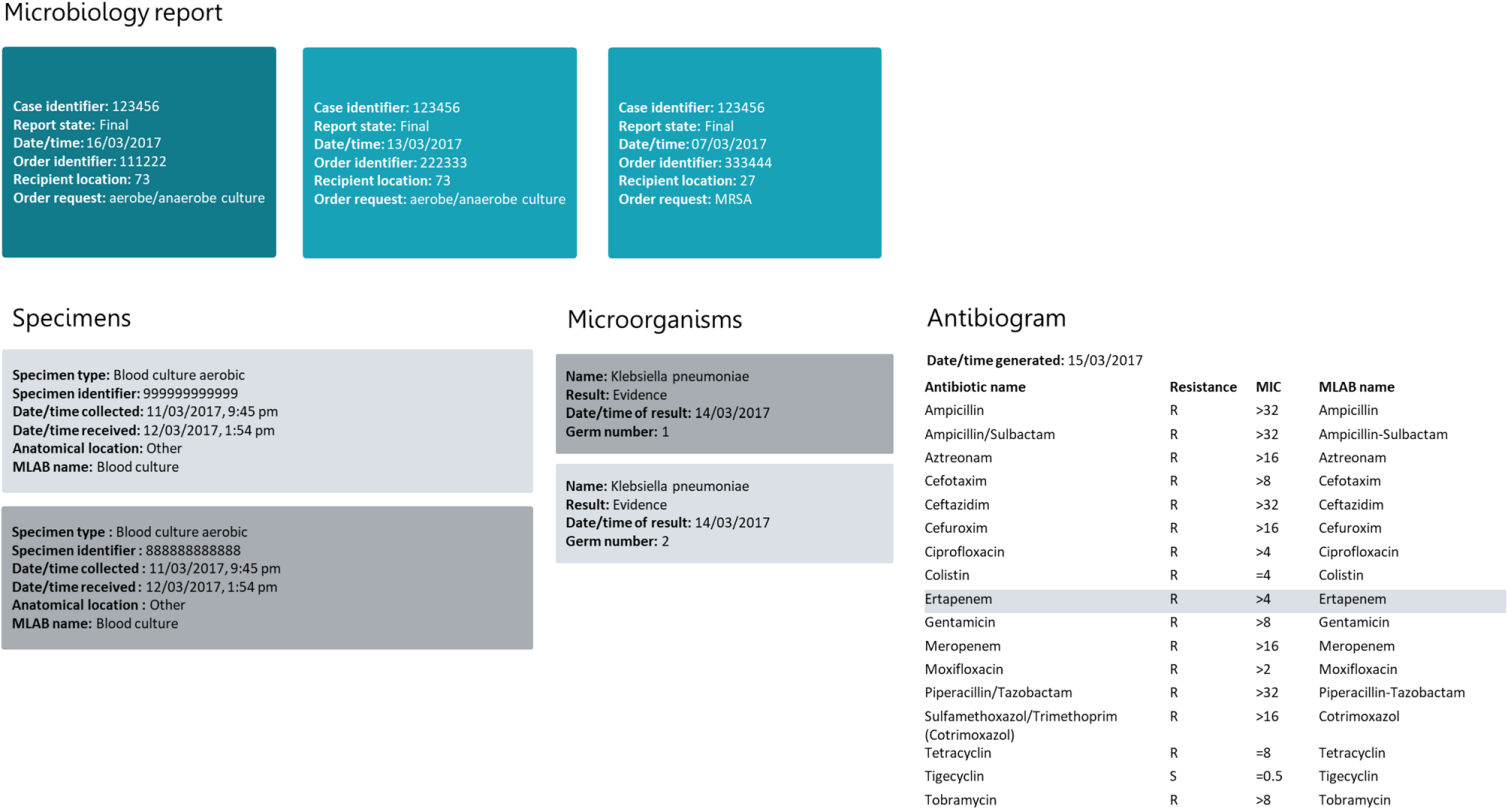
Microbiology report of the selected encounter, visualised in the openMibi application (1) Overview of three microbiology reports with case identifier, report state, date/time, order identifier, recipient location, order request, (2) specimens contained in selected report with specimen type, specimen identifier, date/time collected, date/time received, anatomical location, (3) microorganism found with species identification, result, date/time of result, bacterial count, (4) corresponding antibiogram with date/time generated, standardized antibiotic name, resistance, minimum inhibitory concentration and the original antibiotic substance name in the primary system.

Based on the platform specification, we designed an openEHR based application for microbiology data (‘openMibi’) relying on querying relevant data from the standardized repository by AQL queries (see Supplementary Information, Supplementary Data 3). The application was created with Angular, version 9.0.0. Queries to the openEHR platform are done via REST using AQL. It also offers a prototypical graphical user interface for linking clinical and administrative data (e. g. patient hospital stays and microbiological findings). A patient can be found by entering a unique identifier called EHR-ID (electronic health record identifier) that is linked with the unique patient identifier at the hospital. All encounters, for which microbiological findings exist, can be selected from a dropdown list. If an encounter is selected, the corresponding microbiological findings are displayed. Associated specimens, bacteria and antibiograms are displayed step-by-step depending on selection (see Fig. 4). At each step, a new AQL based query is sent to the platform, whereby the query parameters are dynamically modified depending on what was selected in the previous step (see Supplementary Information, Supplementary Data 4).

To illustrate some of the other possibilities for querying and analysing the platforms’ data as well, we prepared three instructive step-by-step examples, which can be found in the Supplementary Information: (i) using AQL to identify blood culture samples with more than one bacterial species (see Supplementary Data 5), (ii) using the ‘openMibi’ application with a query builder tool to browse through all *Klebsiella pneumoniae* results that are resistant to Ciprofloxacin (see Supplementary Data 6) and (iii) using another self-developed application called ‘openCQA’ to visualise the distribution of minimal inhibitory concentrations in a given sample of *Klebsiella pneumoniae* isolates (see Supplementary Data 7).

## Evaluation

### Comparison of microbiology reports

This part of the evaluation comprised the comparison of ten transformed microbiology reports with the original reports, including all their relevant entries such as microorganisms (here bacteria), samples and antibiograms. By using the openMibi application, technical experts visualised all transformed data entries related to a pre-defined list (see an example in the Supplementary Information, Supplementary Data 1). The results were compared to the original data sets from the primary source system by clinical experts. By this, the feasibility of the transformation process into a standardized openEHR based representation and its accuracy from a qualitative perspective can be evaluated.

The only discrepancies found within the comparison between the openEHR based representation in the openMibi application and the experts’ documentations were:

- In three out of ten reports, body sites (e.g. right shoulder) seemed to be missing.
- In one out of ten transformed reports, the report state value was incorrect (‘final’ instead of ‘pre-final’).

These aspects were identified as transformation problems which were successfully solved afterwards:

- The body site element was not included in the extraction process because it was not queried from the original database. The item is included and mapped to the corresponding archetype element now.
- A wrong mapping between specific abbreviations of entries describing the state of the report in the primary source system (e.g. ‘F’ for final) caused issues. The mapping was corrected.

### Data quality check

The Supplementary Information, Supplementary Data 8–10 shows some selected general numbers as well as the answers to the pre-defined questions calculated based on the integrated Hannover data sets from two years and the openCQA tool. Overall, our quality check shows that we are not only able of transferring the original data sets into an interoperable format but also that these data sets are correct from a domain expert perspective when looking at some statistics automatically calculated based on these newly formatted data sets.

## Discussion

In this work, we presented an approach for transforming microbiology data from commercial primary source systems into standardized, consented data models by using openEHR and international terminologies. We were able to develop robust, fully open, standardised and, thus, interoperable data models for microbiology data. The publicly available data models can be used across institutions to reliably transform real-life heterogeneous microbiology reports into standardised representations. The implementation of a proof-of-principle application and the evaluation of correctness and data quality demonstrated that the new formats as well as the integration processes are feasible. Thereby, we laid the foundation for developing cross-institutional, automated outbreak detection systems such as the smart infection control system (SmICS). Moreover, our approach allows that every single patient with the associated bacterial species, including the antimicrobial resistance profiles, remains traceable during data analysis and does not get lost in aggregated epidemiologic data.

Computerized and automated infection control efforts have been increasingly described in the recent years for the surveillance of healthcare-associated infections or monitoring of clusters/outbreaks using for instance the WHONET software^34–36^. Leclère et al. systematically reviewed different methods and algorithms for the detection of hospital outbreaks^37^. The authors conclude that valid epidemiological reference standards are necessary to compare different approaches; however they do believe that these novel tools can be useful for infection control professionals to make decisions^37^. The availability of such tools is of enormous importance as nosocomial transmissions in hospitals are an important contributor to the epidemic of MDRO^1^. Schröder et al. analysed different mathematical algorithms for automated outbreak detection and compared the findings with the results of their manual outbreak detection protocol. They succeeded promising results for outbreaks with sporadic bacteria but stress that that further work is necessary when focusing on endemic, i.e. more common, bacteria^7^.

Since detailed standardized data models for the microbiology area are rarely available, the openEHR modelling of an entire microbiology report required great efforts. It took over 250 content review rounds of about 150 international and national reviewers to form the microbiology openEHR data models. Since this was a very time-consuming task, the data models are highly valuable now: as presented in this publication, we are now able to transfer data from its relational and technology-dependent representation into an interoperable, standardized and open data representation, enriched with existing terminologies such as SNOMED CT. Furthermore, this model is of high importance because it is the consented representation of microbiology reports decided by various domain experts from different university medical centres in Germany which joined forces.

We successfully demonstrated the feasibility of designing open, interoperable applications based on this new representation of microbiology data. The graphical representation of the integrated reports allows a quick and comfortable look into the integrated data. By this, errors that may have occurred during the data integration could be revealed immediately. The openMibi app fully relies on the aforementioned standardized data models, value sets and terminology bindings so that all institutions using the same data definitions will be able to reuse this application. Consequently, it can serve as a prototype for a future advanced automatic detection system called smart infection control system (SmICS). Such application will need to take data from other source systems such as administrative and movement data (e. g. admission, discharge, patient’s location during the hospital stay) into account. The focus of this paper were set on the microbiology content needed, however, data models for these categories also have been consented already. Also, the presented openMibi app already includes prototypical functions for querying and integrating both, the administrative and microbiology data, from the same data repository. However, these data models and algorithms are in need of more review rounds before being able to implement them in a fully SmICS application.

The modelling process is a collaborative and cross-institutional task, whereas the data integration workflow depends on the local primary source systems and their database structure. In this publication, we exemplary demonstrated the data integration processes in Hannover. Here, we experienced some yet unknown challenges, e.g. some items for the susceptibility result were not covered in the consented value sets of the microbiology template. Here, close cooperation between clinicians/microbiologists and scientists/data stewards is needed to define a correct mapping to the value sets of the final model. These are typical issues each institution might face in various depths. It is always recommended trying to map the local data sets to the items of the openEHR template before modifying the template to fit the local use case. Sometimes this could not be avoided because currently not all value sets are fully standardised, e.g. the list of microorganisms mapped to standardised terminologies is restricted to a well-known and important list. However, some local terms might not be available in this set, so that it has to be integrated in its current shape without further standardization to assure that nothing will get lost. As soon as these items will get some standardisation, e.g. a SNOMED CT code, it is recommended to update the integrated data sets.

In terms of the evaluation results, we found some discrepancies, which were solved quickly, e.g. a wrong mapping of the original report state values to the defined list (pre-final, final report) or missing entries for the body site from which the specimen was taken. Overall, our quality check shows that we are not only able of transferring the original data sets into an interoperable format but also that these data sets are correct from a domain expert perspective. However, we would like to underline that initial and continuous quality control procedures need to be implemented in the routine workflow to capture potential discrepancies. One reason for a new inconsistency during the routine workflow might be a change in the primary laboratory information system. In fact, the involved data stewards and clinical microbiologists need to be in close contact when system changes or updates take place. These controls can be supported from the technical perspective by storing the original content of each specific data item within the transformed data set. Therefore, we use the so called ‘feeder audit’ class of the openEHR reference model^38^.This feature allows the original content item itself to be directly included or pointed to so that both values can be easily compared.

Furthermore, to enable the quality checks, it is possible to establish a loop that transforms integrated data back into the original representation. Currently, we are working on this procedure and already evaluated it successfully in other settings^39^.

It is essential to establish repeated controls of selected microbiology reports by clinical microbiologists in cooperation with the data stewards. Two of such quality control procedures are described in our manuscript: (1) comparing the raw and the transformed microbiology reports by experts and (2) comparing known (historic) analyses (e.g. antimicrobial resistance statistics or infection statistics) for a given patient population with the analysis results and statistics calculated based on the transformed data sets. Looking at the proportion of MRSA and Meropenem-resistant *Klebsiella pneumoniae* strains in the dataset analysed we found that the results were to some extent higher when comparing to the ‘official’ in-house historic counts for that time period. This might be caused by not implementing copy strain elimination or stratification (e.g. clinical specimens versus screening specimens) yet at this stage. However, algorithms and rules for copy strain elimination will be part of the SmICS application and can be customized to meet local definitions and needs. Furthermore, we experienced that the resistance of an isolate found is not always reported in a separate antibiogram but sometimes in a text block (either relating to another related isolate with an antibiogram or formulating the resistance characteristics in text). Currently, we are not able to access these text blocks so that this also affects our statistics. Nonetheless, the results are quite in the same range on the ‘official’ in-house counts which demonstrates a robust and general feasibility. In terms of the general numbers, we identified a wide range of microbiology reports per patient. For most patients only few microbiology reports are generated, whereas for some a considerably higher amount is available. The maximum of 350 is not a single outlier.

One major challenge is that highly relevant information in the microbiologic results are represented in additional text blocks, e.g. the MDRO class or specific resistance attributes (such as the detection of carbapenemases in Gram-negative bacteria). In the direct future work, we will focus on integrating these text blocks into standardized pattern so that they can be assessed properly. Here, natural language processing (NLP) algorithms can be integrated^40^. In fact, this will be crucial for the outbreak detection as additional relevant information for cluster detection is documented here.

As mentioned above, some models and value list are still highly discussed because no (inter)national concerted models are available up to now. The optimization of these resources is ongoing work, e.g. a request already was made to SNOMED International to add codes for the missing concepts of bacteria we identified. Indeed, there have already been two requests, one for bacteria/organisms and one for specimen concepts. Seven concepts (3 organisms, 1 finding, 3 specimens) were added so far. Two of the requested specimen concepts are already part of the international version of July 2020.

In future, we will roll out some small applications, e.g. openMibi and openCQA, to the other institutions following the openEHR approach and using the defined models to demonstrate the cross-institutional reuse of such open systems in the microbiology area. Afterwards, we will implement and roll out the full SmICS application.

## Conclusions

Automated clinical decision-support systems for infection control promise to be very helpful for nosocomial outbreak detection and may explicitly support infection control professionals in their daily working routine. This is of even higher importance in time of infection control staff shortage. Holistic standardisation of microbiological data is crucial for such systems when operating in a cross-institutional setting and is feasible using an open source approach with openEHR and international terminologies in a real-life multicentre setting.

## Supplementary Information

Supplementary Information.

## Data Availability

The datasets supporting the conclusions of this article are included within the article and its additional files. All data models used can be found at https://ckm.highmed.org/ckm.

## Abbreviations

AQL: Archetype Query Language
ADL: Archetype Definition Language
CDSS: Clinical Decision-Support System
CKM: Clinical Knowledge Manager
ETL: Extraction, Transformation, Load
SmICS: Smart Infection Control System
MeDIC: Medical Data Integration Centre
MDRO: Multidrug resistant organisms
MRSA: Methicillin-resistant *Staphylococcus aureus*
VRE: Vancoymcin resistant enterococci
