# Supplementary material for: Transformation of microbiology data into a standardised data representation using OpenEHR
Source: Sci Rep. 2021 May 18;11:10556. doi: 10.1038/s41598-021-89796-y (PMC8131366; doi:10.1038/s41598-021-89796-y)
Supplement: Supplementary file 1 — Supplementary Information. [file 41598_2021_89796_MOESM1_ESM.pdf]

Transformation of Microbiology Data into a Standardised Data Representation using OpenEHR

Antje Wulff[1*#], Claas Baier[2*#], Sarah Ballout[1], Erik Tute[1], Kim Katrin Sommer[1], Martin Kaase[3], Anneka Sargeant[4], Cora Drenkhahn[5], Infection Control Study Group[6], Dirk Schlüter[2], Michael Marschollek[1#], Simone Scheithauer[3#]

[1]*Peter L. Reichertz Institute for Medical Informatics of TU Braunschweig and Hannover Medical School, Carl-Neuberg-Str. 1, 30625 Hannover, Germany*

[2]*Institute for Medical Microbiology and Hospital Epidemiology, Hannover Medical School, Carl-Neuberg-Str. 1, 30625 Hannover, Germany*

[3]*Institute of Infection Control and Infectious Diseases, University Medical Center Göttingen (UMG), Georg-August University Göttingen, Robert-Koch-Str. 40, 37075 Göttingen, Germany.*

[4]*Institute of Medical Informatics, University Medical Center Göttingen (UMG), Georg-August University Göttingen Robert-Koch-Str. 40, 37075 Göttingen, Germany*

[5]*IT Center for Clinical Research (ITCR-L) and Institute of Medical Informatics (IMI), University of Lübeck, Ratzeburger Allee 160, 23538 Lübeck, Germany*

[6]*List of study group members can be found in the main manuscript.*

[#]these authors *contributed equally* to the work

**Corresponding author at:*

Peter L. Reichertz Institute for Medical Informatics of
TU Braunschweig and Hannover Medical School,
Carl-Neuberg-Str. 1, 30625 Hannover, Germany.
E-Mail address: antje.wulff@plri.de (A.Wulff).
URL: http://www.plri.de (A. Wulff)

**Corresponding author at:*

Institute for Medical Microbiology and Hospital Epidemiology,
Hannover Medical School,
Carl-Neuberg-Str. 1, 30625 Hannover, Germany.
E-Mail address: baier.claas@mh-hannover.de (C. Baier)

Supplementary Information

Supplementary Data 1: Exemplary comparison sheet for microbiology reports 3
Supplementary Data 2: Overview of the value lists defined and mapped onto terminologies* 10
Supplementary Data 3: OpenMibi application 12
Supplementary Data 4: Query parameters for a selected encounter 13
Supplementary Data 5: Retrieving Data from the OpenEHR-based Data Repository by AQL 14
Supplementary Data 6: Retrieving Data from the OpenEHR-based Data Repository by openMibi 20
Supplementary Data 7: Retrieving Data from the OpenEHR-based Data Repository by openCQA 23
Supplementary Data 8: Results of the data quality check with pre-defined tasks and an extract of three exemplary result sets with different complexity levels of the openCQA tool 28
Supplementary Data 9: Automated data quality check: openCQA result (Distribution of microbiology reports) 30
Supplementary Data 10: Automated data quality check: openCQA result (Distribution of bacterial counts) 31

Supplementary Data 1: Exemplary comparison sheet for microbiology reports

Exemplary detailed comparison for the retrospective review of microbiology reports (evaluation)

Microbiology Report for Patient #12345*		
**Criteria**	**OpenEHR Representation**	**Gold Standard Decision**
**Result report available?**	Yes ☒ No ☐	Yes ☒ No ☐
**Report state**	Preliminary	Final
**Report time**	24.05.2018	24.05.2018
**Receiving laboratory location**	Ward 81	Ward 81
**Total number of specimens in the entire report**	1	1
**Specimen #1**		
**Specimen type**	Urine (permanent catheter)	Urine (permanent catheter)
**Total number of the pathogens in the specimen**	2	2
**Culture**	Aerobic and anaerobic culture	Aerobic and anaerobic culture
**Specimen identifier**	123456789	123456789
**Date/time of sampling**	22.05.2018 20:54	22.05.2018 20:54
**Body site**	n/a	left
**Pathogen #1**		
**Pathogen name**	Escherichia coli	Escherichia coli
**Pathogen quantity**	10^5/ml	10^5/ml
**Pathogen #2**		
**Pathogen name**	Enterococcus faecalis	Enterococcus faecalis
**Pathogen quantity**	10^5/ml	10^5/ml

Antibiogram for Pathogen #1		
**Which germ relates to the Antibiogram?**	Specimen #1 Urine (permanent catheter) Pathogen #1 (Eschrichia coli)	Specimen #1 Urine (permanent catheter) Pathogen #1 (Eschrichia coli)
**Antibiotic name**	Ampicillin	Ampicillin
**MIC value**	>= 32	>=32
**Susceptibility**	R ☒ I ☐ S ☐	R ☒ I ☐ S ☐
**Antibiotic name**	Ampicillin/Sulbactam	Ampicillin-Sulbactam
**MIC value**	>= 32	>=32
**Susceptibility**	R ☒ I ☐ S ☐	R ☒ I ☐ S ☐
**Antibiotic name**	Piperacillin/Sulbactam	Piperacillin Sulbactam
**MIC value**	-	-
**Susceptibility**	R ☒ I ☐ S ☐	R ☒ I ☐ S ☐
**Antibiotic name**	Piperacillin/Tazobactam	Piperacillin-Tazobactam
**MIC value**	>= 128	>=128
**Susceptibility**	R ☒ I ☐ S ☐	R ☒ I ☐ S ☐
**Antibiotic name**	Cefuroxim	Cefuroxim
**MIC value**	= 16	16
**Susceptibility**	R ☒ I ☐ S ☐	R ☒ I ☐ S ☐
**Antibiotic name**	Cefuroxime-Axetil	Cefuroxime-Axetil
**MIC value**	16	16
**Susceptibility**	R ☒ I ☐ S ☐	R ☒ I ☐ S ☐
**Antibiotic name**	Cefpodoxim	Cefpodoxim
**MIC value**	= 2	2
**Susceptibility**	R ☒ I ☐ S ☐	R ☒ I ☐ S ☐

**Antibiotic name**	Ceftriaxon	Ceftriaxon
**MIC value**	-	-
**Susceptibility**	R ☐ I ☐ S ☒	R ☐ I ☐ S ☒
**Antibiotic name**	Cefotaxim	Cefotaxim
**MIC value**	<= 1	<=1
**Susceptibility**	R ☐ I ☐ S ☒	R ☐ I ☐ S ☒
**Antibiotic name**	Ceftazidim	Ceftazidim
**MIC value**	<= 1	<=1
**Susceptibility**	R ☐ I ☐ S ☒	R ☐ I ☐ S ☒
**Antibiotic name**	Gentamicin	Gentamicin
**MIC value**	<= 1	=1
**Susceptibility**	R ☐ I ☐ S ☒	R ☐ I ☐ S ☒
**Antibiotic name**	Tobramycin	Tobramycin
**MIC value**	-	-
**Susceptibility**	R ☐ I ☐ S ☒	R ☐ I ☐ S ☒
**Antibiotic name**	Fosfomycin	Fosfomycin
**MIC value**	<= 16	<=16
**Susceptibility**	R ☐ I ☐ S ☒	R ☐ I ☐ S ☒
**Antibiotic name**	Nitrofurantoin	Nitrofurantoin
**MIC value**	<= 16	<=16
**Susceptibility**	R ☐ I ☐ S ☒	R ☐ I ☐ S ☒
**Antibiotic name**	Levofloxacin	Levofloxacin
**MIC value**	<= 0.12	<=0.12
**Susceptibility**	R ☐ I ☐ S ☒	R ☐ I ☐ S ☒
**Antibiotic name**	Ciprofloxacin	Ciprofloxacin
**MIC value**	<= 0.25	<=0.25

**Susceptibility**	R ☐ I ☐ S ☒	R ☐ I ☐ S ☒
**Antibiotic name**	Moxifloxacin	Moxifloxacin
**MIC value**	-	-
**Susceptibility**	R ☐ I ☐ S ☒	R ☐ I ☐ S ☒
**Antibiotic name**	Meropenem	Meropenem
**MIC value**	<= 0.25	<=0.25
**Susceptibility**	R ☐ I ☐ S ☒	R ☐ I ☐ S ☒
**Antibiotic name**	Sulfamethoxazol/Trimethoprim (Cotrimoxazol)	Cotrimoxazol
**MIC value**	>= 320	>=320
**Susceptibility**	R ☒ I ☐ S ☐	R ☒ I ☐ S ☐
**Antibiogram 2**		
**Which germ relates to the Antibiogram?**	Specimen #1 Urine (permanent catheter) Pathogen #2 (Enterococcus faecalis)	Specimen #1 Urine (permanent catheter) Pathogen #2 (Enterococcus faecalis)
**Antibiotic name**	Ampicillin	Ampicillin
**MIC value**	<= 2	<=2
**Susceptibility**	R ☐ I ☐ S ☒	R ☐ I ☐ S ☒
**Antibiotic name**	Ampicillin/Sulbactam	Ampicillin-Sulbactam
**MIC value**	<= 2	<=2
**Susceptibility**	R ☐ I ☐ S ☒	R ☐ I ☐ S ☒
**Antibiotic name**	Gentamicin High-Level	Gentamicin (Hochresistenz)
**MIC value**	-	-
**Susceptibility**	R ☐ I ☐ S ☒	R ☐ I ☐ S ☐
**Antibiotic name**	Doxycyclin	Doxycycline
**MIC value**	-	-
**Susceptibility**	R ☒ I ☐ S ☐	R ☒ I ☐ S ☐
**Antibiotic name**	Tetracyclin	Tetracyclin

**MIC value**	>= 16	>=16
**Susceptibility**	R ☒ I ☐ S ☐	R ☒ I ☐ S ☐
**Antibiotic name**	Tigecyclin	Tigecyclin
**MIC value**	<=0.12	<0.12
**Susceptibility**	R ☐ I ☐ S ☒	R ☐ I ☐ S ☒
**Antibiotic name**	Nitrofurantoin	Nitrofurantoin
**MIC value**	<= 16	<=16
**Susceptibility**	R ☐ I ☐ S ☒	R ☐ I ☐ S ☒
**Antibiotic name**	Levofloxacin	Levofloxacin
**MIC value**	= 1	1
**Susceptibility**	R ☐ I ☐ S ☒	R ☐ I ☐ S ☒
**Antibiotic name**	Imipenem	Imipenem
**MIC value**	<= 1	<=1
**Susceptibility**	R ☐ I ☐ S ☒	R ☐ I ☐ S ☒
**Antibiotic name**	Vancomycin	Vancomycin
**MIC value**	= 2	2
**Susceptibility**	R ☐ I ☐ S ☒	R ☐ I ☐ S ☒
**Antibiotic name**	Teicoplanin	Teicoplanin
**MIC value**	<= 0.5	<0.5
**Susceptibility**	R ☐ I ☐ S ☒	R ☐ I ☐ S ☒
**Antibiotic name**	Sulfamethoxazol/Trimethoprim (Cotrimoxazol)	Cotrimoxazol
**MIC value**	<= 10	<=10
**Susceptibility**	R ☐ I ☒ S ☐	R ☐ I ☒ S ☐
**Antibiotic name**	Quinupristin/Dalfopristin	Quinu-/Dalfopristin
**MIC value**	= 8	8
**Susceptibility**	R ☒ I ☐ S ☐	R ☒ I ☐ S ☐

**Antibiotic name**	Linezolid	Linezolid
**MIC value**	= 2	2
**Susceptibility**	R ☐ I ☐ S ☒	R ☐ I ☐ S ☒

Table 1: Exemplary comparison sheet for microbiology reports

Supplementary Data 2: Overview of the value lists defined and mapped onto terminologies*

Archetype	Item	Consented value list	Terminology
**Result report**	State	Preliminary report, final report	Local terms
**Laboratory test**	Test name	Microbiology examination [fixed value]	Local terms
**Specimen**	Specimen type	Blood culture aerobic, blood culture anaerobic, blood culture, swab<br>+ *free text for additional values*	SNOMED CT
**Anatomical location**	Body site name	Anus, nose, nasopharyngeal, throat, rectum, arterial, venous<br>+ *free text for additional values*	SNOMED CT
**Laboratory test analyte**	Analyte	Presence, Absence	Local terms
**Laboratory test analyte**	Analyte result (identified pathogen)	List available in CKM: https://ckm.highmed.org/ckm/document?cid=1246.159.96	SNOMED CT
**Pathogen details**	Frequency	List available in CKM: https://ckm.highmed.org/ckm/document?cid=1246.159.101	SNOMED CT
**Pathogen details – slot antibiogram**	Antibiotic name	List available in CKM: https://ckm.highmed.org/ckm/document?cid=1246.159.98	LOINC
**Pathogen details – slot antibiogram**	Susceptibility	Resistant, Susceptible (standard dosing regimen), Susceptible (increased exposure)	e.g. EUCAST
**Pathogen details**	MDRO class	e.g. MRSA, VRE	Local terms, based on national definitions

**Laboratory test**	Order request	List available in CKM: https://ckm.highmed.org/ckm/document?cid=1246.159.97 + *free text for additional values*	LOINC

Table 2: Overview of archetypes used for representing microbiology data

*To outsource the complexity of terminology interaction from within applications, the HiGHmed consortium decided to license a terminology server: *Ontoserver* by CSIRO (*doi: 10.1186/s13326-018-0191-z*)

Supplementary Data 3: OpenMibi application

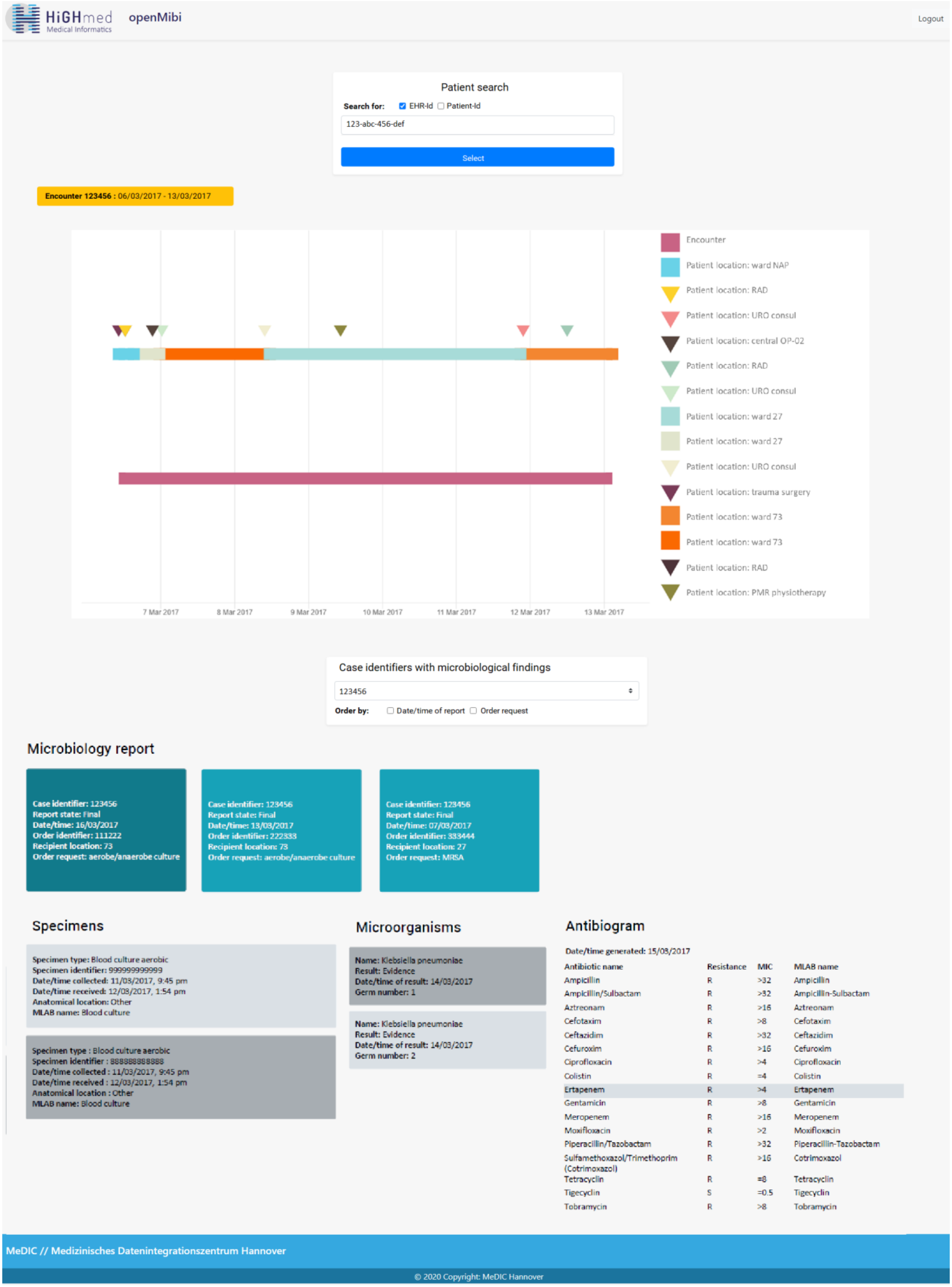

Figure 1: Full view of the openEHR-based application for microbiology data (openMibi)

Supplementary Data 4: Query parameters for a selected encounter

Query parameters for a selected encounter (openMibi)

Number	Section	Items displayed	AQL parameter
**(1)**	Microbiology Report	Encounter identifier, status, date/time, order-ID, recipient location, order request	EHR-ID<br>Encounter-ID
**(2)**	Specimens	Specimen type, specimen identifier, date/time collected, date/time received, anatomical location	EHR-ID<br>Report-ID
**(3)**	Microoganism	Name, result, date/time of result, germ number	EHR-ID<br>Report-ID<br>Specimen-ID
**(4)**	Antibiograms	Date/time generated, antibiotic name, resistance, minimum inhibitory concentration	EHR-ID<br>Report-ID<br>Specimen-ID<br>Pathogen-ID

AQL, Archetype Query Language

Table 3: Query parameters for a selected encounter in the openMibi application

Supplementary Data 5: Retrieving Data from the OpenEHR-based Data Repository by AQL

Example:

Find all patients with a blood culture sample carrying more than one species

Based on the standardized data repository many different data queries can be tailored and specified by the user. Therefore, the archetype query language (AQL)[1] can be used. AQL is a query language that acts directly upon the openEHR-based data model (called *archetype*) and not the database level meaning that each AQL query is executable in any openEHR based data repository as long as the same archetypes are used.

<u>Step 0: Check availability of data in openEHR based format and access rights and credentials</u>

Data must be integrated into and available from an openEHR based data repository (e. g. *Better Platform* by Better d.o.o. in Ljubljana[2], SL or *Ehrbase* (open source), commercial support by Vitasystems in Mannheim, DE[3]) at each institution, according to the consented and standardized data models. The user who would like to access data needs to gain access rights, credentials and connection parameters (e.g. the link to the query editor of the chosen repository or the link to a specific REST API).

<u>Step 1: Identification of the openEHR template representing the data required</u>

In our case, we are looking for blood culture samples and microbiology results, so we need to search data in the data model *microbiology report*[4].

---

[1] http://www.openehr.org/releases/QUERY/latest/docs/AQL/AQL.html
[2] https://platform.better.care/
[3] https://ehrbase.org/
[4] http://ckm.highmed.org/ckm/templates/1246.169.69

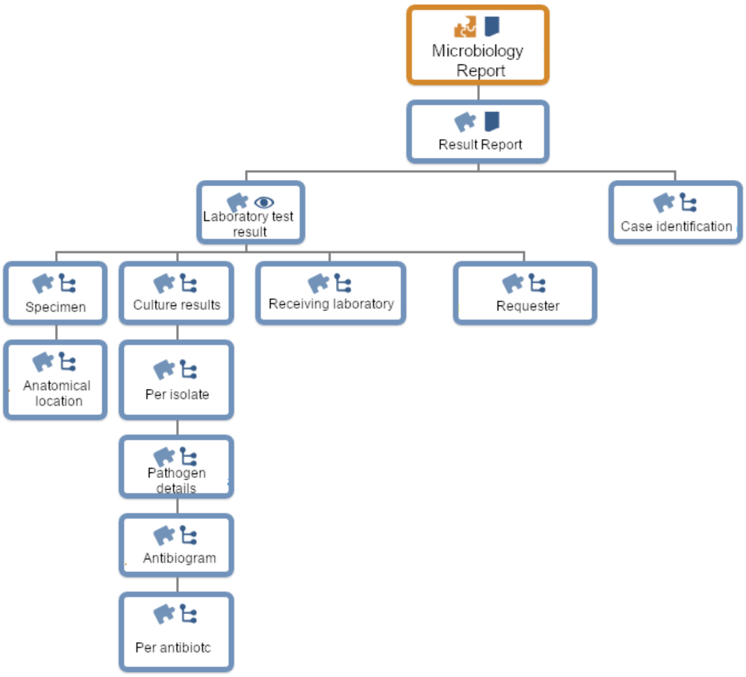

Figure 2: Overview of the hierarchy structure of the openEHR template for microbiology results

<u>Step 2: Identification of the data items needed</u>

According to our example, we need the (1) patient identifiers as the result of our query. With these identifiers, we can further look for more details of each patient (which is not included in this example).

However, we would like to find patients with specific microbiology results. Consequently, we also need data items referring to (2) an existing blood culture sample (data item: *specimen type* of the archetype specimen[5]), and (3) a culture result (data item: *analyte result* [identified pathogen] of the archetype laboratory analyte result[6]). To find out whether the blood culture sample delivers one or more results, we are also interested in the (4) *specimen identifier* (data item: laboratory specimen identifier of the archetype specimen).

<u>Step 3: Reproduce template hierarchy in query</u>

All relevant data items are included in the above-mentioned template. For the query, we need to reproduce the template hierarchy (Figure 2) in the query as needed for the search request.

To look for microbiology reports in the repository independent of a specific patient, we need to specify in the query that a so called 'composition' with the name

---

[5] https://ckm.highmed.org/ckm/archetypes/1246.145.748

[6] https://ckm.highmed.org/ckm/archetypes/1246.145.396

‘microbiology report’ must be available in any electronic health record (*contains composition microbiology report*).

Since we are searching for samples with microorganisms found, we need to specify that the report contains a sample (*contains cluster specimen*) and a finding (*contains cluster culture result*). As you can see in Figure 2, the specimen and the culture results are nested into another element called ‘laboratory test result’ which is why we need to include this above those two ‘contains’ statements as follows:

```
[…]
EHR e
CONTAINS COMPOSITION c[openEHR-EHR-COMPOSITION.report-
result.v1,’Microbiology report']
  CONTAINS (
     CLUSTER d[openEHR-EHR-CLUSTER.specimen.v1]
       AND
     OBSERVATION o[openEHR-EHR-OBSERVATION.laboratory_test_result.v1]
         CONTAINS CLUSTER w[openEHR-EHR-CLUSTER.laboratory_test_analyte.v1]
          )
```

Step 4: Find the correct technical path pointing to the data items

A patient can be identified by the so-called *ehr-id* representing an unique identifier of every patient. The corresponding so-called path is *e/ehr_id/value*.

Each archetype item is identified by a unique path that can be accessed to retrieve data from this archetype. The corresponding paths can be looked up for instance in the CKM (German) [7]. Clicking on ‘Show Paths’ visualizes an unique path to the specific data item.

The required paths for this search request are:

```
   (1)   e/ehr_id/value as patient_id
   (2)   d/items[at0001]/value/id as specimen_id
   (3)   d/items[at0029]/value/value as specimen_type
   (4)   w/items[at0001]/value/value as pathogen_identified
```

The first letter points to the corresponding archetype carrying this data item (see above, e.g. specimen comes with the variable ‘d’ which is why the path to *specimen_id* stats with *d/*).

[7] http://ckm.highmed.org/ckm/templates/1246.169.69

Step 4: Create SELECT FROM statement

It is now possible to create the query using the SELECT FROM statement as known from other query languages.

'DISTINCT' can be used to assure that now duplicates are retrieved.

```
SELECT DISTINCT
e/ehr_id/value as patient_id,
d/items[at0001]/value/id as specimen_id,
d/items[at0029]/value/value as specimen_type,
w/items[at0001]/value/value as pathogen_identified
FROM EHR e
CONTAINS COMPOSITION c[openEHR-EHR-COMPOSITION.report-
result.v1,'Microbiology report']
      CONTAINS (
          CLUSTER d[openEHR-EHR-CLUSTER.specimen.v1]
          AND
          OBSERVATION o[openEHR-EHR-OBSERVATION.laboratory_test_result.v1]
          CONTAINS CLUSTER w[openEHR-EHR-
CLUSTER.laboratory_test_analyte.v1]
          )
```

Step 5: Limit results

We are only interested in blood culture samples and in microbiology results delivering one or more microorganisms found which is why we can add a 'WHERE' statement in which such limiting requirements can be defined as follows:

```
WHERE
exists w/items[at0001]/value/value and
d/items[at0029]/value matches {'blood culture aerobic', 'blood culture
anaerobic', 'blood culture'}
```

Step 6: Sort results

The results can be ordered by the specimen id and the specimen type to identify whether two or more results were found in a specific specimen.

```
ORDER BY
order by d/items[at0001]/value/id, d/items[at0029]/value/value asc
```

Step 7: Finalize query and send to repository

The final query for instance can look like this:

```
SELECT DISTINCT
e/ehr_id/value as patient_id,
d/items[at0001]/value/id as specimen_id,
d/items[at0029]/value/value as specimen_type,
w/items[at0001]/value/value as pathogen_identified
FROM EHR e
CONTAINS COMPOSITION c[openEHR-EHR-COMPOSITION.report-
result.v1,'Microbiology report']
      CONTAINS (
          CLUSTER d[openEHR-EHR-CLUSTER.specimen.v1]
          AND
          OBSERVATION o[openEHR-EHR-OBSERVATION.laboratory_test_result.v1]
          CONTAINS CLUSTER w[openEHR-EHR-
CLUSTER.laboratory_test_analyte.v1]
          )
WHERE
exists w/items[at0001]/value/value and
d/items[at0029]/value matches {'blood culture aerobic', 'blood culture
anaerobic', 'blood culture'}
ORDER BY
order by d/items[at0001]/value/id, d/items[at0029]/value/value asc
```

There are different ways to send this query to the data repository according to the repository chosen and the connection approaches (see step 0). Since these are technical issues, we abstain from presenting these.

A result might look like this (in a table format):

patient_id	specimen_id	specimen_type	pathogen_identified
12345	00001	blood culture aerobic	Staphylococcus epidermidis
12345	00002	blood culture anaerobic	Staphylococcus epidermidis
678910	00003	blood culture aerobic	Micrococcus luteus
111213	00004	blood culture aerobic	Staphylococcus epidermidis
111213	00005	blood culture anaerobic	Staphylococcus epidermidis
141516	00006	blood culture aerobic	Staphylococcus capitis
141516	00007	blood culture anaerobic	Staphylococcus capitis
171819	00008	blood culture aerobic	Streptococcus anginosus
171819	00008	blood culture aerobic	Pediococcus pentosaceus
171819	00009	blood culture anaerobic	Staphylococcus epidermidis

Table 4: AQL result (table format)

By looking at these structured results, it is easy to identify all patients with a blood culture sample carrying more than one species. However, there are a lot more possibilities to define queries, e.g. you can also work with so-called 'GROUP BY' and 'COUNT' statements. By this, it is also possible to identify all patients with a blood culture sample carrying more than one species fully automatically. These queries

come with an increased complexity, which is why we decided to present the above-given simplified query instead.

Supplementary Data 6: Retrieving Data from the OpenEHR-based Data Repository by openMibi

Example:

Find all *Klebsiella pneumoniae* isolates resistant to Ciprofloxacin

Specific applications can be developed to interact with the data repository. An example is our self-developed tool called openMibi. We included a simple query builder to illustrate the capabilities of such tools for retrieving data as specified by a user through such application.

Step 0: Check availability of data in openEHR based format and access rights and credentials for openMibi

Data must be integrated into and available from an openEHR based data repository (e. g. *Better Platform* by Better d.o.o. in Ljubljana, SL[8] or *Ehrbase* (open source), commercial support by Vitasystems in Mannheim, DE[9]) at each institution, according to the consented and standardized data models. The user who would like to access data needs to gain access rights, credentials and connection parameters (e.g. the link to the openMibi tool).

Step 1: Open tool

OpenMibi is a web-based tool that can be accessed via a specific link when installed at the participating institution.

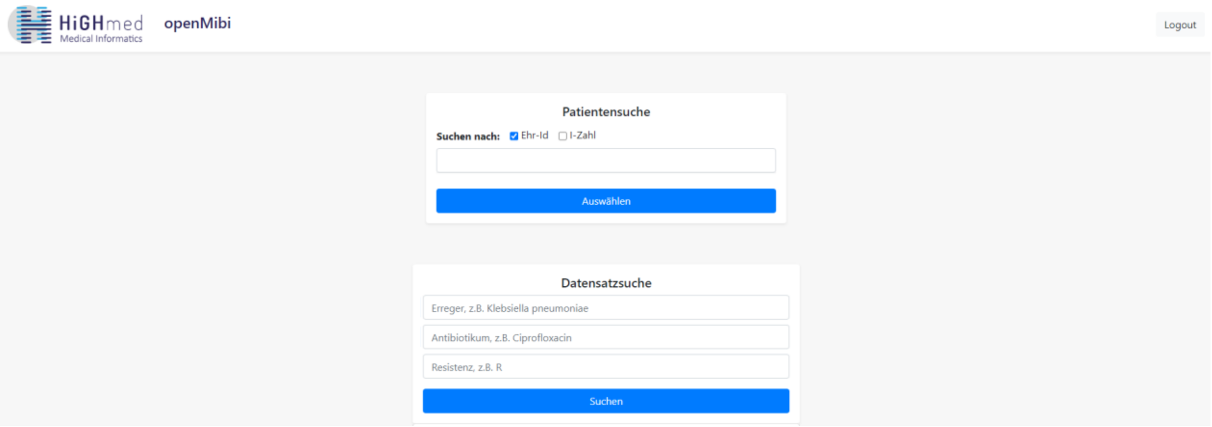

Figure 3: OpenMibi

[8] https://platform.better.care/
[9] https://ehrbase.org/

Step 2: Configure parameters

The user can either retrieve all microbiology reports by searching for a specific patient (as explained in Supplementary Information xx) or by using the newly added exemplary query builder. The user can define the desired microorganisms of interest, the antibiotic name and the resistance.

Step 3: Creating query

In the background, the application now creates a query based on the archetype query language (AQL) and sends it to the repository.

Step 4: Visualisation of results

The openMibi tool now is able of visualising all microbiology reports by their unique identifiers according to this request.

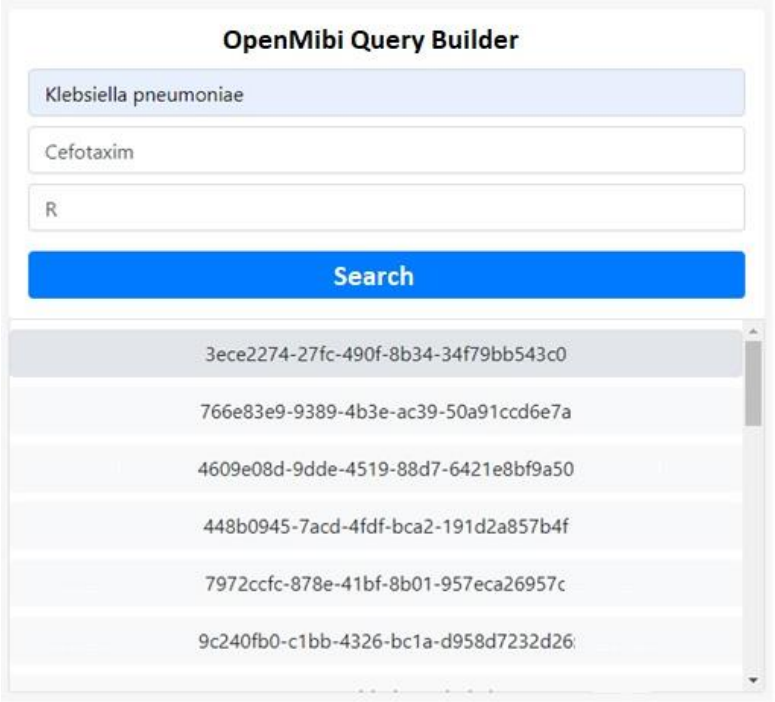

Figure 4: OpenMibi Query Builder with first results

Step 5: Browsing through reports

By clicking on a specific report identifier, the full report will be visualised as presented in Supplementary Data 3 and can be browsed.

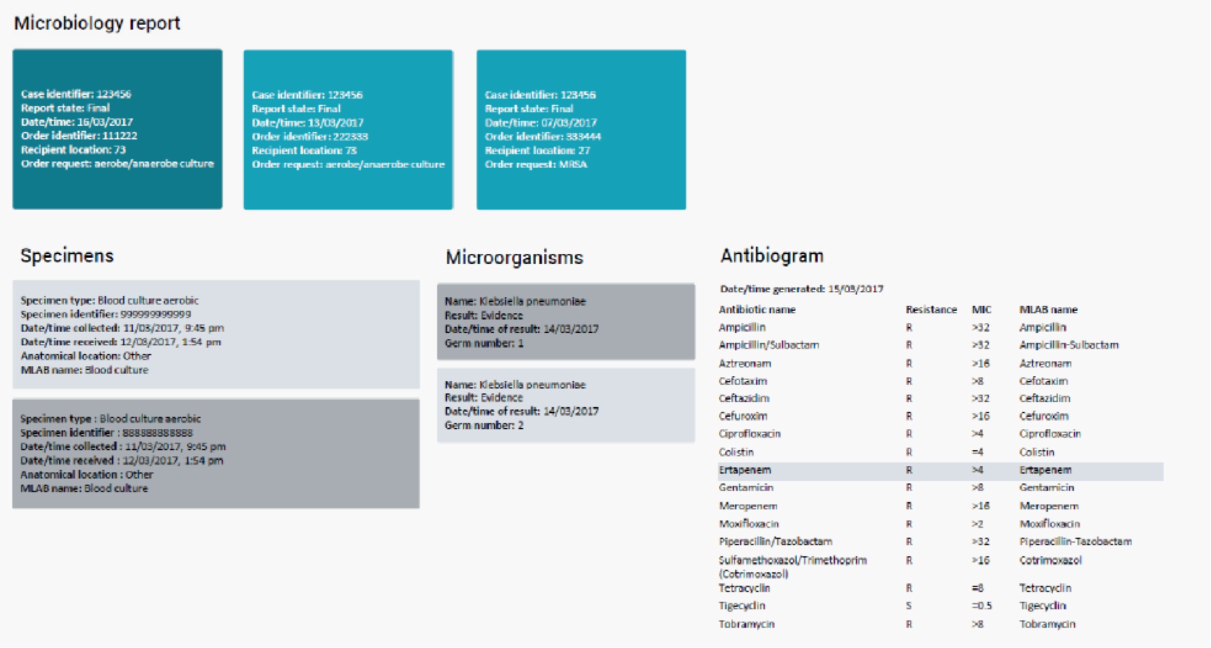

Figure 5: OpenMibi – visualisation of microbiology report

Supplementary Data 7: Retrieving Data from the OpenEHR-based Data Repository by openCQA

Example:

MIC distribution of Ciprofloxacin and Cefotaxim in *Klebsiella pneumoniae* isolates from patients in 01/2017- 06/2017

Another example on how users can interact with the standardized data repository through an application can be visualized with our self-developed openCQA tool[10]. Here, the user needs to specify a query that delivers all potential relevant data sets. After that, the user can apply different statistical calculations that are automatically proposed by the tool. In the end, a specific analysis, such as the distribution of MIC for specific antibiotics and microorganisms (e.g. *MIC distribution of Ciprofloxacin and Cefotaxim in Klebsiella pneumoniae isolates from patients in 01/2017-06/2017*) are visualized.

<u>Step 0: Check availability of data in openEHR based format and access rights and credentials for openCQA</u>

Data must be integrated into and available from an openEHR based data repository (e. g. *Better Platform* by Better d.o.o. in Ljubljana[11], SL or *Ehrbase* (open source), commercial support by Vitasystems in Mannheim, DE[12]) at each institution, according to the consented and standardized data models. The user who would like to access data needs to gain access rights, credentials and connection parameters (e.g. the link to the openCQA tool).

<u>Step 1: Open tool</u>

OpenCQA is a web-based tool that can be accessed via a specific link when installed at the participating institution.

[10] https://pubmed.ncbi.nlm.nih.gov/33750371/
[11] https://platform.better.care/
[12] https://ehrbase.org/

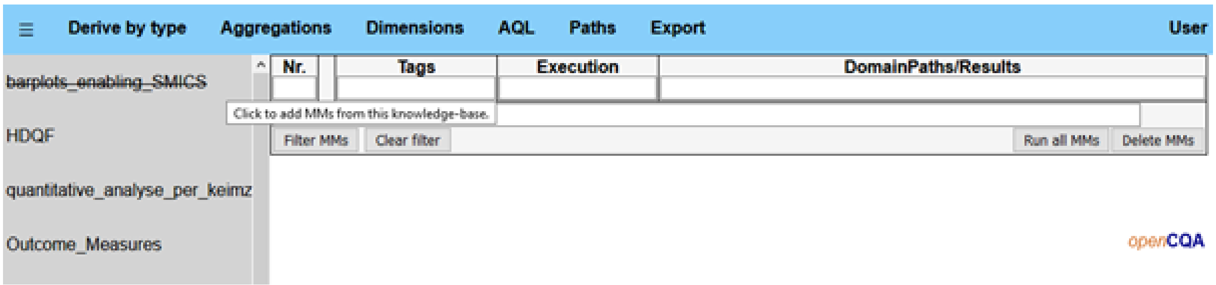

Figure 6: OpenCQA

Step 2: Create query

By clicking on ‘AQL’, the user can define an AQL query to load data into the tool.

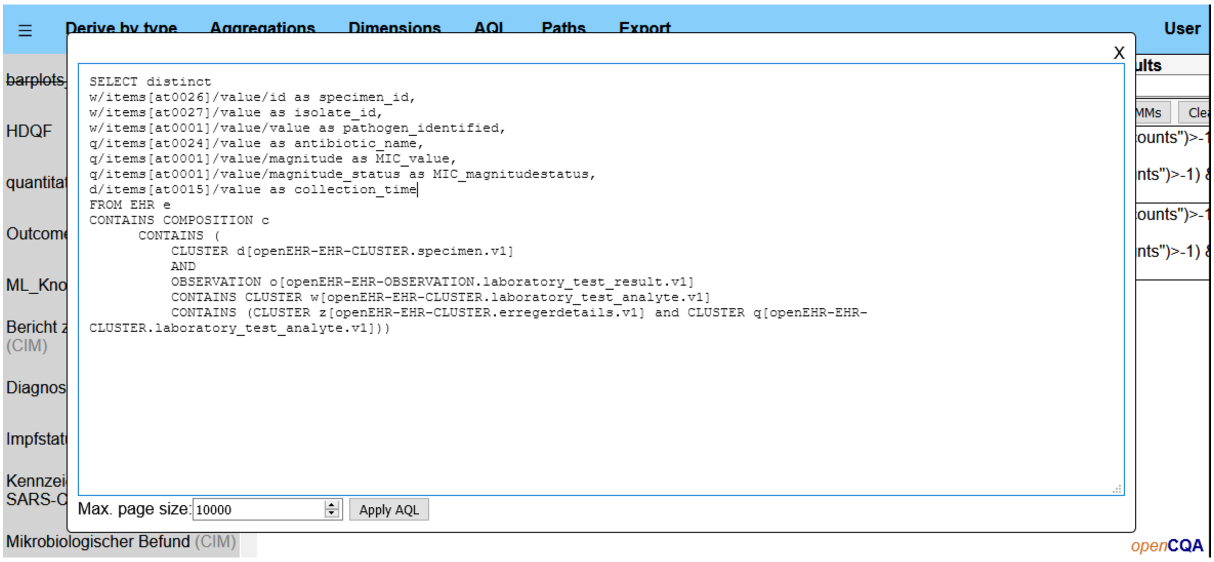

Figure 7: OpenCQA - AQL builder

Step 3: Choose pre-defined methods

By clicking on ‘derive by type’, a set of pre-defined statistical methods are loaded and can be chosen (e.g. count, min, max, median).

Step 4: Edit pre-defined methods

By clicking on ‘edit’, the pre-defined methods can be edited and customized. For instance, in ‘group’, a grouping rule can be specified to reach a ‘count per group’ as needed in our example. Furthermore, the ‘characterization’ input field can be filled with any R code to define new functions or methods. In our case, we wrote a new method to visualise the results as a customized bar plot.

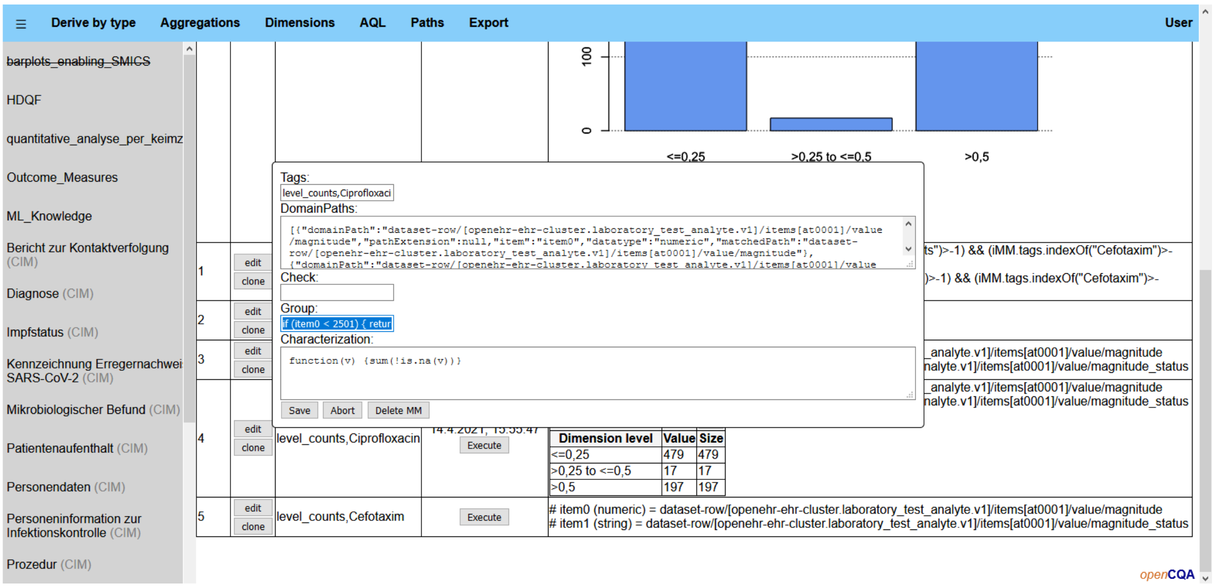

Figure 8: OpenCQA – Edit pre-defined methods

Alternative step for 2-4

For reoccurring statistical tasks or calculations, all of the above-mentioned procedures can be stored in a so-called 'knowledge base'. They are directly loaded when starting openCQA.

Step 5: Visualisation of results

By clicking on 'execute', the results for the chosen or edited methods are visualized.

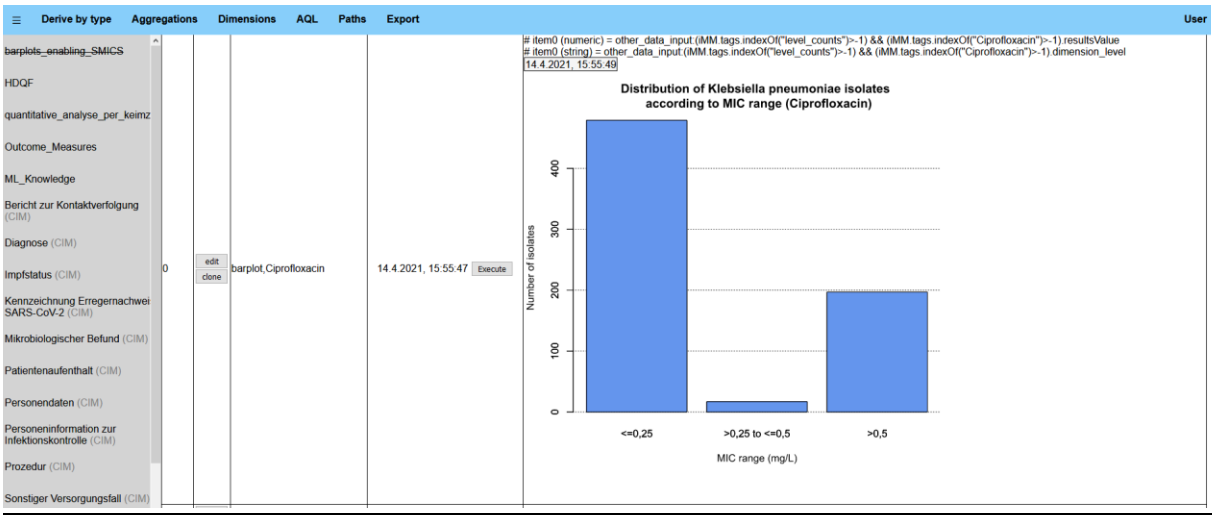

Figure 9: OpenCQA – Results (1)

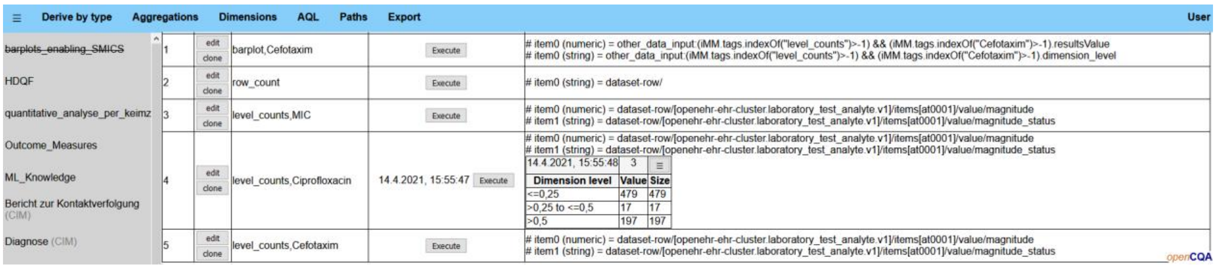

Figure 10: OpenCQA – Results (2)

The results for our example look like this:

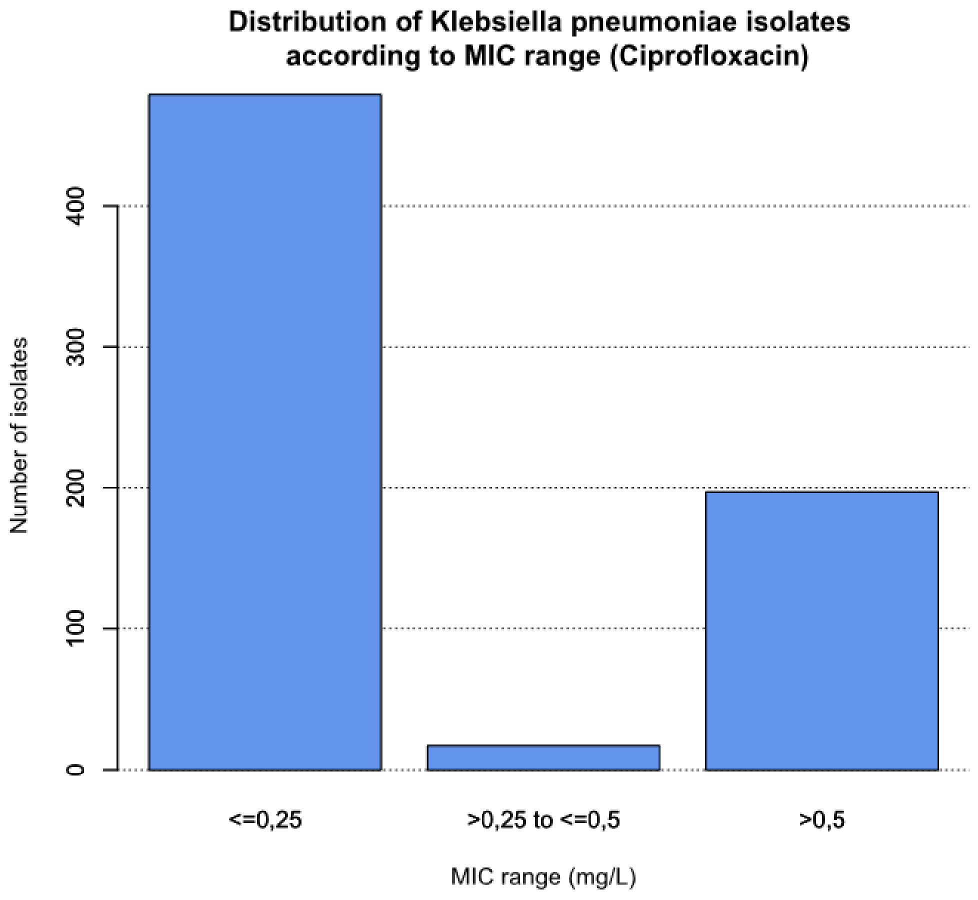

Figure 11: Distribution of *Klebsiella pneumoniae* isolates according to MIC range (Ciprofloxacin)

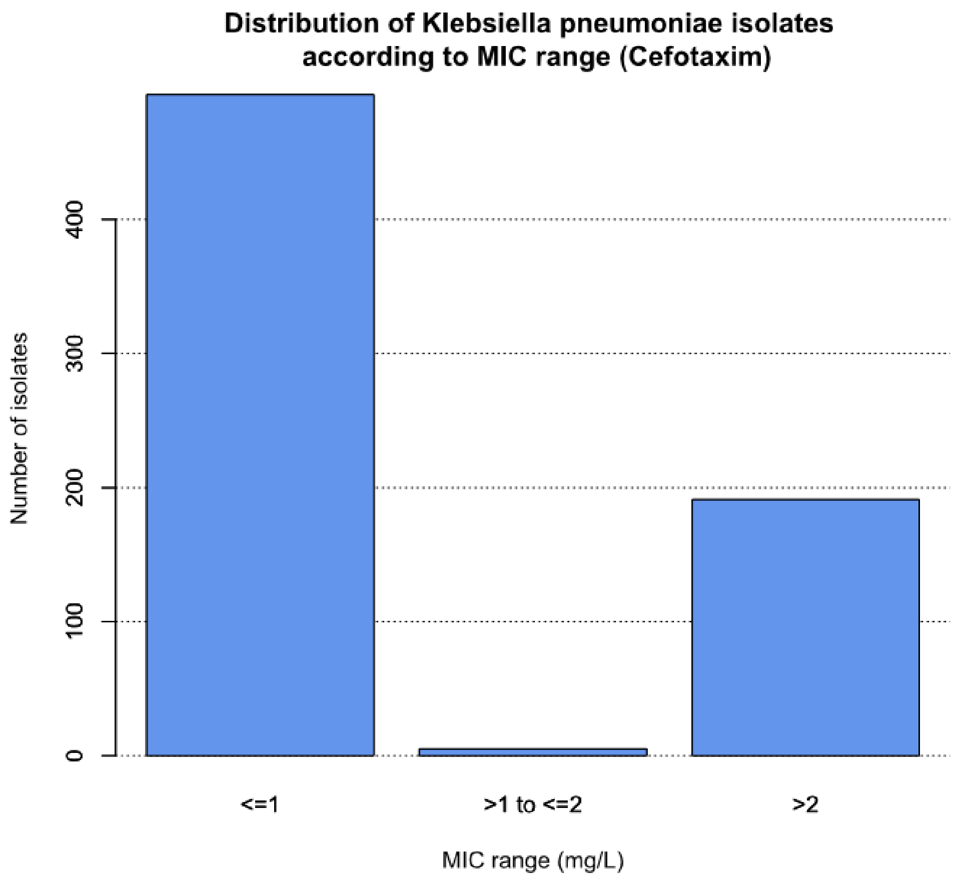

Figure 12: Distribution of *Klebsiella pneumoniae* isolates according to MIC range (Cefoxtaxim)

Supplementary Data 8: Results of the data quality check with pre-defined tasks and an extract of three exemplary result sets with different complexity levels of the openCQA tool

<table>
<tr><th>Task</th><th>openCQA Result</th></tr>
<tr><td>General information</td><td>
<table>
<tr><th>Dimension</th><th>Count</th></tr>
<tr><td>Patients</td><td>51947</td></tr>
<tr><td>Microbiology reports</td><td>260.084</td></tr>
<tr><td>Specimens</td><td>303344</td></tr>
<tr><td>Species</td><td>754</td></tr>
<tr><td>Isolates</td><td>240594</td></tr>
<tr><td>Antibiograms</td><td>103742</td></tr>
</table>
<u>Distribution of microbiology reports per patient (see Supplementary Data 9 for a box plot)</u>
<table>
<tr><td>Mean</td><td>5.006718</td></tr>
<tr><td>Median</td><td>2</td></tr>
<tr><td>Maximum</td><td>350</td></tr>
</table>
</td></tr>
<tr><td>Proportion of MRSA</td><td><u>Percentage of MRSA on all Staphylococcus aureus isolates</u>
Number of Staphylococcus aureus isolates: 7916
Number of Methicillin-resistant Staphylococcus aureus isolates: 1724
= 1724/7916 = 0.218 = 21.8% (including copy strains)</td></tr>
<tr><td>Proportion of Meropenem-resistant Klebsiella pneumoniae</td><td>Number of Klebsiella pneumoniae isolates: 3367
Number of Meropenem-resistant Klebsiella pneumoniae isolates: 164
= 164/3367 = 0.049 = 4.9% (including copy strains)</td></tr>
</table>

<table>
<tr><td>Quantitative analysis</td><td><u>Distribution of bacterial counts in positive urine samples with Escherichia coli as the sole microorganism (see Supplementary Data 10 for the diagram):</u>
<table>
<tr><th>Bacterial count</th><th>Frequency</th></tr>
<tr><td>not specified</td><td>242</td></tr>
<tr><td>10^2/ml</td><td>258</td></tr>
<tr><td>10^3/ml</td><td>477</td></tr>
<tr><td>10^4/ml</td><td>985</td></tr>
<tr><td>10^5/ml</td><td>3,638</td></tr>
</table></td></tr>
<tr><td>Top 10 of isolates found per species (with antibiogram)</td><td>
<table>
<tr><th>Species</th><th>Number of isolates</th></tr>
<tr><td>Escherichia coli</td><td>11246</td></tr>
<tr><td>Staphylococcus aureus</td><td>7916</td></tr>
<tr><td>Pseudomonas aeruginosa</td><td>7775</td></tr>
<tr><td>Enterococcus faecium</td><td>4818</td></tr>
<tr><td>Enterococcus faecalis</td><td>4483</td></tr>
<tr><td>Klebsiella pneumoniae</td><td>3367</td></tr>
<tr><td>Staphylococcus epidermidis</td><td>3274</td></tr>
<tr><td>Enterobacter cloacae</td><td>2143</td></tr>
<tr><td>Klebsiella oxytoca</td><td>1740</td></tr>
<tr><td>Proteus mirabilis</td><td>1677</td></tr>
</table></td></tr>
</table>

Table 5: Results of the data quality check with pre-defined tasks and an extract of three exemplary result sets with different complexity levels of the openCQA tool

Supplementary Data 9: Automated data quality check: openCQA result (Distribution of microbiology reports)

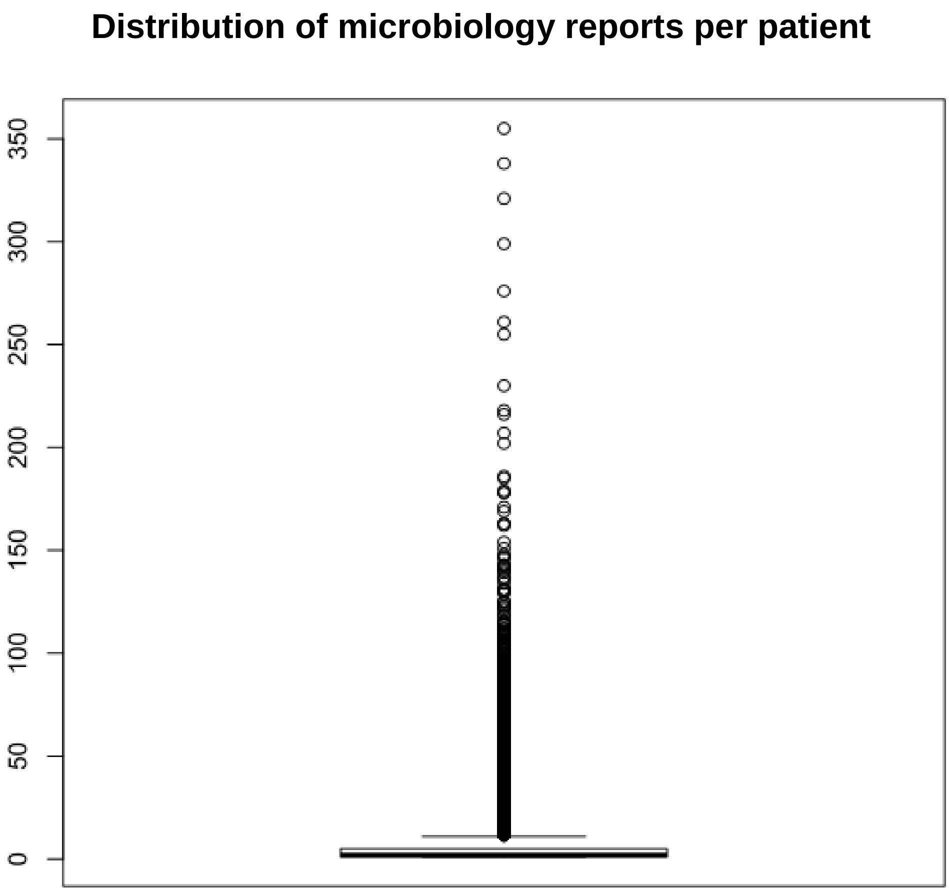

Figure 13: Distribution of microbiology reports per patient

Supplementary Data 10: Automated data quality check: openCQA result (Distribution of bacterial counts)

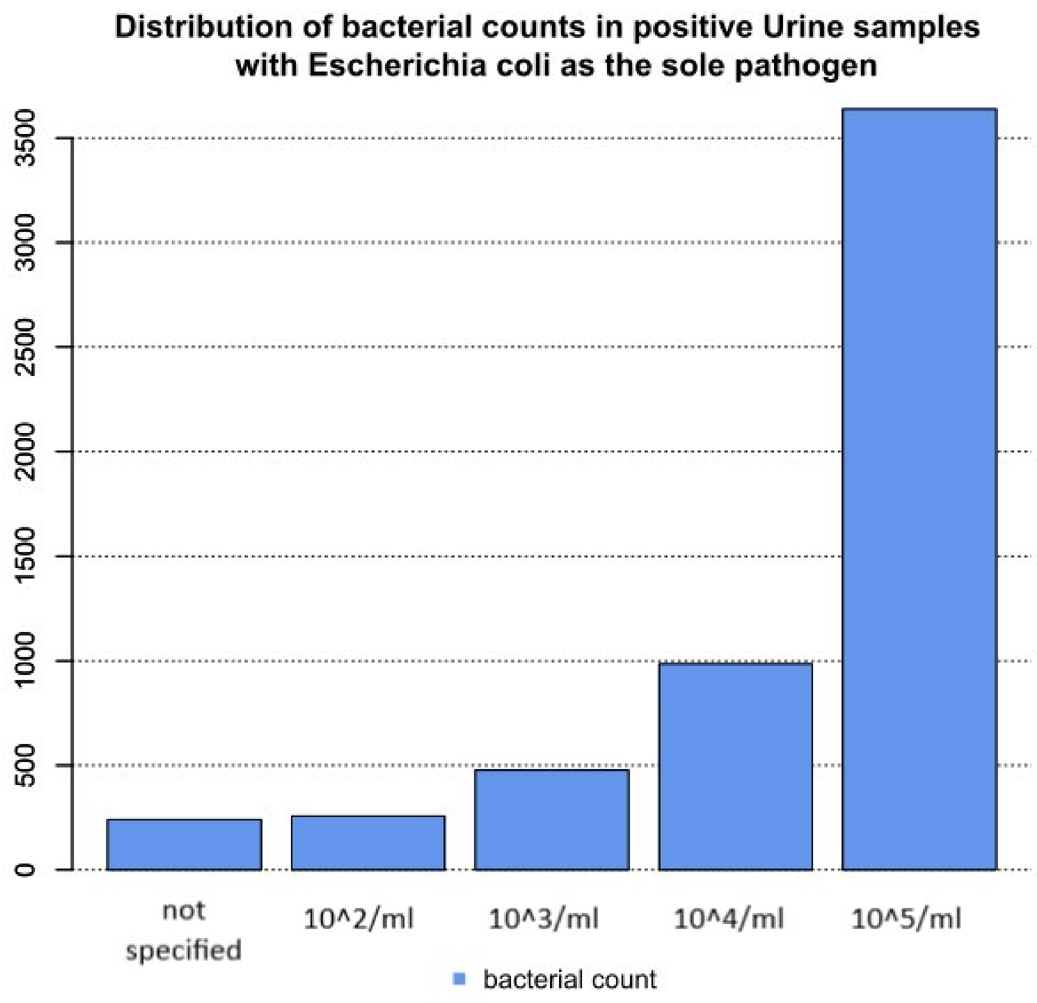

Figure 14: OpenCQA result on distribution of bacterial counts in positive Urine samples with Escherichia coli as the sole bacteria
